# A Nudibranch Marine Extract Selectively Chemosensitizes Colorectal Cancer Cells by Inducing ROS-Mediated Endoplasmic Reticulum Stress

**DOI:** 10.3389/fphar.2021.625946

**Published:** 2021-04-08

**Authors:** Verónica Ruiz-Torres, Nicholas Forsythe, Almudena Pérez-Sánchez, Sandra Van Schaeybroeck, Enrique Barrajón-Catalán, Vicente Micol

**Affiliations:** ^1^ Instituto de Investigación, Desarrollo e Innovación en Biotecnología Sanitaria de Elche (IDiBE), Universitas Miguel Hernández (UMH), Elche, Spain; ^2^ Drug Resistance Group, Centre for Cancer Research and Cell Biology, School of Medicine, Dentistry and Biomedical Science, Queen’s University Belfast, Belfast, United Kingdom; ^3^ CIBER, Fisiopatología de la Obesidad y la Nutrición, CIBERobn, Instituto de Salud Carlos III, Mallorca, Spain

**Keywords:** marine compounds, stress balance, antiproliferative activity, colon cancer, marine biotechnology and natural products

## Abstract

The present study shows the putative antiproliferative mechanism of action of the previously analytically characterized nudibranch extract (*Dolabella auricularia,* NB) and its different effects in colon cancer cells vs. nontumor colon cells. NB extract increased the accumulation of reactive oxygen species (ROS) and increased endoplasmic reticulum (ER) stress via stimulation of the unfolded protein response. Stress scavengers, N-acetylcysteine (NAC) and 4-phenylbutyric acid (4-PBA), decreased the stress induced by NB. The results showed that NB extract increased ER stress through overproduction of ROS in superinvasive colon cancer cells, decreased their resistance threshold, and produced a nonreturn level of ER stress, causing DNA damage and cell cycle arrest, which prevented them from achieving hyperproliferative capacity and migrating to and invading other tissues. On the contrary, NB extract had a considerably lower effect on nontumor human colon cells, suggesting a selective effect related to stress balance homeostasis. In conclusion, our results confirm that the growth and malignancy of colon cancer cells can be decreased by marine compounds through the modification of one of the most potent resistance mechanisms present in tumor cells; this characteristic differentiates cancer cells from nontumor cells in terms of stress balance.

## Introduction

Oceans cover 70% of the planet’s surface and represent more than 90% of the habitats involved in the formation of life. The marine ecosystem is a hostile environment where mostly sessile or slowly moving species compete to survive and protect themselves by developing defensive chemical weapons (or secondary metabolites). Bioactive marine compounds have considerable diversity and complexity and have attracted scientific interest in many fields; specifically, marine compounds have become a new potential source of anticancer substances (Ruiz-Torres et al., 2017; University, 2020).

Cancer is a worldwide leading cause of death; colorectal cancer (CRC) is the third most common cancer in men, second most common cancer in women (Colorectal Cancer, 2018), and first most common cancer when both sexes are considered together. This study focuses on the changes in metabolic scenarios in CRC cells compared to normal colon cells.

Cancer cells have important redox deregulation, resulting in persistent high intracellular levels of reactive oxygen species (ROS), which have been defined by many studies to be tumor-promoting agents (Kim et al., 2016). The ability of tumor cells to survive at high levels of oxidative stress (OS) is based on their improved antioxidant defense system (Davison et al., 2013). Overactivation of the nicotinamide adenine dinucleotide phosphate– (NADPH-) and glutathione- (GSH-) related antioxidant enzymes and cofactors, such as nicotinamide adenine dinucleotide (NADH) and flavin adenine dinucleotide (FADH), and the overexpression of the transcription factor Nrf2 have been reported to contribute to the balance of OS in cancer cells. Furthermore, these antioxidant enzymes can facilitate multiple mechanisms to trigger the spread of metastatic cancer cells (Kumari et al., 2018). Piskounova et al. (Piskounova et al., 2015) demonstrated that metastatic cells have stronger antioxidant properties than nonmetastatic cells, which allow them to survive at a high level of OS.

As a result of the uncontrolled growth of cancer cells and the role of the endoplasmic reticulum (ER) in the synthesis and folding of proteins, ER plays an important role in cancer progression. Changes in the cellular environment, such as OS generation, DNA damage, nutrient deprivation, hypoxia, and calcium depletion, can compromise ER homeostasis, inducing the accumulation of improperly folded proteins. When misfolded proteins inside the ER lumen are accumulated and the ER protein folding capacity is overwhelmed, ER stress is produced. In this situation, the initial survival adaptation response of cells is called the unfolded protein response (UPR), which promotes the reestablishment of ER homeostasis. The UPR is controlled by three ER-transmembrane stress sensors: pancreatic endoplasmic reticulum kinase (PERK), inositol-requiring enzyme 1α (IRE1α), and activating transcription factor 6 (ATF6). When ER stress is chronically prolonged and exceeds its fold capacity, cellular dysfunction and cell death are induced (Riha et al., 2017).

ER stress and OS are closely linked and result from altered cellular metabolism in various cancer types (Gorlach et al., 2006). In the last few years, the scientific community has been increasingly interested in developing therapies to treat the altered metabolism of tumor cells. The high-stress levels in tumor cells differentiate them from nontumor cells and make them more vulnerable to metabolic modulators that induce cell death (Luengo et al., 2017). Therefore, targeting the ROS and ER stress pathways and determining their relationships with the mechanisms involved in cancer development are new potential strategies to prevent cancer.

**FIGURE 1:**
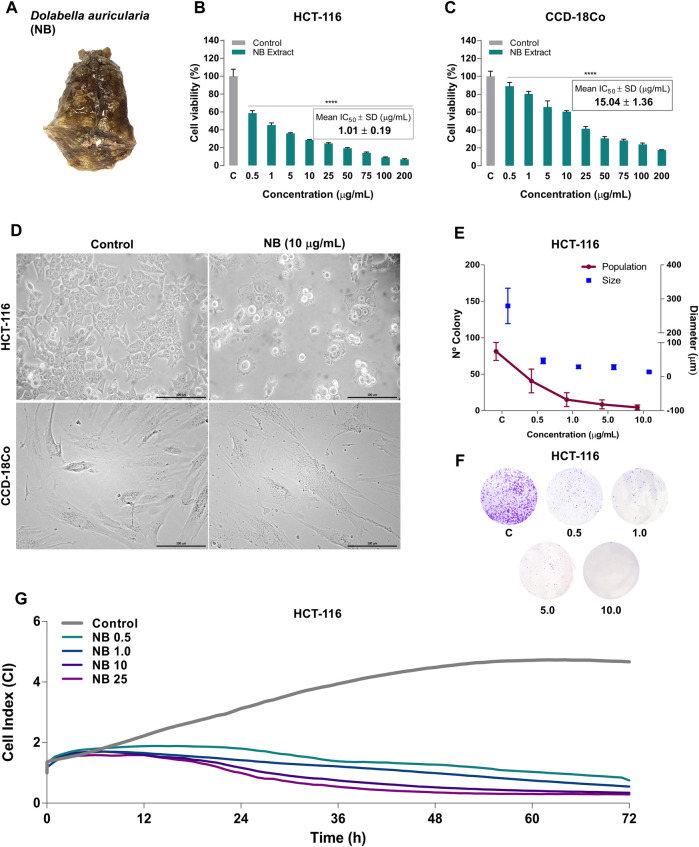
Effect of *Dolabella auricularia* extract (NB) on the proliferation of colon cancer and normal cell lines. **(A)** Photograph of a *Dolabella auricularia* invertebrate specimen. The cytotoxicity of various concentrations of NB extract after 24 h of treatment was tested by the MTT assay in HCT-116 **(B)** and CCD-18Co **(C)** cells. The control (C) is cells plus DMSO (extract vehicle; at less than 0.2% (v:v)). The results are reported as the mean ± SD of three independent experiments. **(D)** Morphology of HCT-116 and CCD-18Co untreated cells (left, control) and cells treated with NB extract (right, 10 µg/ml). **(E)** The survival of HCT-116 cells was measured by the clonogenic assay. The plot shows the size and number of colonies. Data are presented as the mean ± SD of three independent experiments. *p*-values were calculated by comparison with the untreated cell line using ANOVA. *p-value < 0.05, **p-value < 0.01, *** *p*-value < 0.001, and **** *p*-value < 0.0001. **(F)** Colonies stained with crystal violet. **(G)** HCT-116 cell proliferation as the cell index (CIP) of HCT-116 was measured by a real-time cell analyzer using E-plates for 72 h. The mean kinetic curves of three independent experiments are shown.

In a previous study (Ruiz-Torres et al., 2019), a nudibranch extract (*Dolabella auricularia*, NB) (Figure 1A) was selected as the extract with the highest antiproliferative activity after screening twenty extracts in a panel of human CRC cell lines (HGUE-C-1, HT-29, and SW-480). NB was found to be a potent inducer of an imbalance in oxidative homeostasis by increasing intracellular oxygen species (ROS), generating DNA damage, depolarizing mitochondria, and causing cell cycle arrest in the G2/M phase. In the present study, the antiproliferative effect of NB extract was investigated in the clinically relevant colon cancer HCT-116 cell model and compared with the nontumoral human colon cell line CCD-18Co. The relationship between the antiproliferative activity and the increase in ROS and ER stress induced by NB extract was analyzed, with a special interest in the proteins and mechanisms involved in these effects. The antimetastatic effects of NB extract in the highly aggressive and metastatic HCT-116 human CRC cell line (De Toledo et al., 2012; Ahmed et al., 2013) and isolated HCT-116 populations selected based on their enhanced invasiveness were also studied. The results confirm the putative relevance of *D. auricularia* extract in the development of new treatments for CRC management and contribute to the understanding of the mechanism linking the imbalance in stress status with proliferation and metastasis.

## Materials and Methods

### Chemicals and Reagents

Organic solvents for preparing the extract, such as dichloromethane, methanol, and DMSO, labels such as Thiazolyl Blue Tetrazolium Bromide (MTT), Crystal Violet, Hoechst 33342, and 2′,7′-dichlorodihydrofluorescein diacetate (H_2_DCF-DA), and N-acetyl-L-cysteine (NAC), 4-phenylbutyric acid (4-PBA), chloroquine (Chlo), necrostatin-1 (Nec-1), and necrosulfonamide (Nsa) compounds were purchased from Sigma-Aldrich (Europe). Z-VAD-FMK (zVAD) was obtained from APExBIO Technology, United States.

### Preparation of Marine Extract

The *Dolabella auricularia* nudibranch (NB) was provided by the distributor company of marine species Todopez SL (Monforte del Cid, Spain). The fresh organisms were cut into small pieces and extracted with dichloromethane:methanol (D:M) (1:1, v:v) at 4°C for 24 h. After that, the solution was filtered and evaporated to dryness on a rotatory vacuum evaporator, dried by speed vac, and stored at −80°C until use. For *in vitro* culture evaluation of biological activity, NB was dissolved in DMSO at 50 mg/ml. The concentrations of DMSO used did not affect cell viability.

### Cell Culture

Human colorectal carcinoma HCT-116 and fibroblasts from the normal colon (CCD-18Co) were purchased from the American Type Culture Collection (ATCC, MN, United States). HCT-116 cell line was maintained in Dulbecco’s modified Eagle’s medium (DMEM) (Gibco, ThermoFisher, Inc., United States) and normal colon cell line CCD-18Co was cultured in Eagle’s Minimum Essential Medium (ATCC, MN, United States). Both media were supplemented with a 10% fetal bovine serum (FBS), 100 U/mL penicillin, and 100 g/ml streptomycin. Cells were maintained at 37°C in a humidified atmosphere containing 5/95% of CO_2_/air.

### Cell Viability Assay

HCT-116 and CCD-18Co cells were seeded in 96-multiwell culture plates at a density of 5 × 10^3^ cells/well. After 24 h incubation with NB extract at 0.5, 1, 5, 10, 25, 50, 100, and 200 μg/ml, MTT was added (concentration of 0.5 mg/ml).

After 3 h of incubation, the formazan crystals were dissolved in DMSO and absorbance was measured at 570 nm in a microplate reader (SPECTROstar Omega, BMG Labtech, Germany). Cell viability was directly proportional to the absorbance and presented as the mean ± SD from three independent experiments. The cell viability was calculated as the percentage of untreated cells (control cells plus dimethyl sulfoxide less than 0.2%, C) and the IC_50_ values were determined using GraphPad Prism v5.0 software (GraphPad Software, La Jolla, CA, United States).

### Colony Formation Assay

The colony formation assay is based on the ability of a single cell to form a colony (above 50 cells or more). This technique determines the effectiveness of cytotoxic agents (Franken et al., 2006). The HCT-116 cell line (5 × 10^2^ cells per well) was seeded in 6-well plates. After 24 h, when cells were attached to the plate, cells were treated with NB extract at 0.5, 1.0, 5.0, and 10.0 µg/m. After 24 h of treatment, a fresh medium was added and renewed for 14 days for colony formation. Cell nuclei were labeled with Hoechst 33342 and fixed with 95% ethanol for 10 min; then, cells were washed three times with PBS 1X. The number of colonies and their diameters were measured using the Cell Imaging Multi-Mode Reader Cytation 3 (BioTek, Germany). Photos of colonies were taken staining cells with 0.1% crystal violet solution for 10 min; then, cells were washed three times with PBS 1X.

### Proliferation, Migration, and Invasion Assays

For these assays, HCT-116 and HCT-116 invasive populations were used. Superinvasive HCT-116 populations were isolated using Corning^®^ BioCoat™ Matrigel^®^ Invasion Chamber, 8.0 µm PET Membrane 6-well plate (Corning, Europe). This plate contained 2 chambers separated by an insert of 8.0 µm, coated with Matrigel. The plate was prepared with DMEM with 10% FBS (chemoattractant) in the bottom chamber and DMEM without FBS in the upper chamber. 1.25 × 10^5^ HCT-116 parental (*p*) cells/ml were seeded in the upper chamber and allowed to invade the bottom chamber for 48 h. Invasive cells from the bottom chamber were collected and seeded in a 25 cm^2^ flask to get confluence. The process was repeated 4 times to obtain invasive subpopulation 4 (I4) and 9 times to isolate invasive 9 (I9).

The proliferation, migration, and invasion rate were monitored in real-time using the xCELLigence system Real-Time Cell Analyzer (RTCA) (Roche Diagnostics GmbH, Germany). This system registered the impedance values of each well and transformed then into a cell index (CI) value.

For proliferation assay, HCT-116P, I9, and I4 cells were seeded at a density of 7.5 × 10^3^ cells/well in an E-Plate (Roche Diagnostics GmbH, Germany). After 24 h, when the proliferation cell index (CI) was near the value of 1 as manufacturer’s recommendations, cells were treated with NB extract at 0.5, 1, 5, and 10 µg/ml and CI was automatically monitored for the duration of 72 h.

For migration and invasion assay, HCT-116P, I4, and I9 were serum-starved 6 h before conducting the experiment. In the bottom chamber, NB extract at different concentrations in media culture with SFB at 10% were prepared and two controls were added (negative migration control, C-, with 0% SFB; positive migration control, C+, with 10% of SFB). CIM-Plate (Roche Diagnostics GmbH, Germany) was allowed to equilibrate into the incubator for 1 h. 4 × 10^4^ HCT-116 cells were seeded in each upper chamber in serum-free media. The impedance value was monitored for 48 h and expressed as a cell index of migration (CIM).

For invasion assay, the procedure was the same as the migration assay, however, the CIM-Plate (Roche Diagnostics GmbH, Germany) was coated with a 1:40 solution of Matrigel™ (BD Biosciences, United States) according to the manufacturer’s instruction. The impedance value was monitored for 48 h and expressed as a cell index of invasion (CII).

### ROS Detection

Colon cell lines were seeded at 5 × 10^3^/well into 96-well plates and treated with different concentrations of NB extract. After treatment, 2′,7′-dichlorodihydrofluorescein diacetate (H_2_DCF-DA, Sigma-Aldrich, Europe) (10 μM) for intracellular ROS measurement and DNA staining Hoechst 33342 (Sigma-Aldrich, Europe) (10 μg/ml) were incubated for 30 min. Cells were washed with PBS 1X and the fluorescent values (H_2_DCF-DA, Ex/Em: 495/527 nm and Hoechst 33342, Ex/Em: 361⁄497) were obtained using the Cell Imaging Multi-Mode Reader Cytation (BioTek Instruments, Inc., United States). H_2_DCF-DA fluorescent values were normalized to Hoechst fluorescent values. Data of normalized ROS from cells treated with NB extract were statistical compared to untreated cells (control cells plus dimethyl sulfoxide less than 0.2%, C) and UV control (untreated cells irradiated with UVB at 800 J/m^2^).

### ER Membrane Dye

ER membranes were imaged using the cell-permeable live-cell dye ER-Tracker Red (BODIPY TR Glibenclamide) (Invitrogen, ThermoFisher, Inc., USA). HCT-116 cells were seeded at 5 × 10^3^/well into 96-well plates and treated with different concentrations of NB extract. After treatment, ER-Tracker (1 μM) and DNA staining Hoechst 33342 (Sigma-Aldrich) (10 μg/ml) were incubated for 30 min in Hankʼs Balanced Salt Solution with calcium and magnesium (HBSS, Gibco, ThermoFisher, Inc, USA) at 37°C, 5% CO_2_. After incubation, the staining solution was replaced with fresh medium and the fluorescent values of ER-Tracker (587/615 nm) and Hoechst 33342 (361⁄497 nm) were obtained using the Cell Imaging Multi-Mode Reader Cytation (BioTek Instruments, Inc., United States).

### Flow Cytometry Assays (Cell Cycle, Apoptosis, and DNA Damage)

CRC HCT-116 cells were seeded at 1.5 × 10^5^ cells per well into 6-well plates and treated with NB extract at different concentrations for 24 h and then harvested and washed with PBS 1X. Cell cycle, apoptosis by Annexin V method, and DNA damage by H2A.γ method were tested following the manufacturer’s instructions, using the Muse^®^ Cell Analyzer (Merck KGaA, Darmstadt, Germany) and data were analyzed using Muse ^TM^ 1.4 software (Merck KGaA, Darmstadt, Germany).

For cell cycle analysis, cells were fixed with 70% ethanol for at least 3 h prior to staining. Ethanol was removed by centrifuging. Cells were washed with PBS 1X and resuspended in 200 μL of Muse™ Cell Cycle Reagent, which includes RNAse A and the nuclear DNA intercalating stain propidium iodide (PI), and incubated for 30 min at room temperature, protected from light. The suspension sample was analyzed on Muse™ Cell Analyzer, which quantifies the percentage of cells in the G0/G1, S, and G2/M phases of the cell cycle. Flow cytometric analysis collected 10,000 events.

Apoptosis was measured using the Muse™ Annexin V & Dead Cell kit that combines Annexin V to detect phosphatidylserine (PS) on the external membrane of apoptotic cells combined with 7-AAD, a dead cell marker as an indicator of cell membrane structural integrity. A 100 μL of the Muse™ Annexin V & Dead Cell Reagent was added to 100 μL of cells in suspension and mixed and incubated for 20 min at room temperature in the dark. The suspension sample was analyzed on Muse™ Cell Analyzer that leads to distinguish four populations: nonapoptotic cells (Annexin V (−) and 7-AAD (−)), early apoptotic cells (Annexin V (+) and 7-AAD (−)), late-stage apoptotic and dead cells (Annexin V (+) and 7-AAD (+)), and nuclear debris (Annexin V (−) and 7-AAD (+)). Flow cytometric analysis collected 5,000 events.

The DNA damage was detected by using the Muse™ H2A.X Activation Dual Detection Kit. This kit includes two directly conjugated antibodies, a phospho-specific anti-phospho-Histone H2A. γ (Ser139)-Alexa Fluor^®^555 and an anti-Histone H2A. γPECy5 conjugated antibody to measure total levels of Histone H2A. γ. The level of phosphorylated protein and total can be measured simultaneously in the same cell, resulting in a normalized H2A.X activation. Cells were fixed for 5 min on ice and permeabilized by adding Permeabilization Buffer for 5 min and then resuspended in Assay Buffer. Cells were stained by adding 10 µL of antibody working cocktail solution and incubating for 30 min in the dark at room temperature. Cells were washed and analyzed on the Muse™ Cell Analyzer.

### Caspases Detection

For the caspase 8 and 3/7 activity, colon cancer cells were seeded at 2 × 10^5^ cells in a 6-well plate and treated with NB extract for 24 h. Cells were lysed and the amount of protein was determined using Thermo Scientific Pierce Kit (BCA Protein assay kit) at 562 nm. An equal amount of protein was mixed with 100 µL of the Caspase-Glo 3/7 and Caspase-Glo 8 assay kit (Promega, Madison, WI, United States) to each well. The mixture was incubated for 1 h at room temperature for 1 h in the dark. Luminescence was measured using the Cell Imaging Multi-Mode Reader Cytation (BioTek Instruments, Inc., United States).

### Reverse Transfection

HCT-116 cells were transfected with siRNA using Hiperfect (Qiagen N.V., Germany) according to the manufacturer’s reverse-transfection protocol. Scrambled control siRNA and C8 or C9 siRNA were prepared in RNAse-free water (Qiagen N.V., Germany) in 90 dishes to get a final concentration of 10 nM. HiPerfect and Optimem (Gibco, ThermoFisher, Inc., United States) were added and incubated for 30 min at room temperature. Cells were seeded in the mastermix at 1.5 × 10^6^ cells per dish. After 24  h, the culture medium was switched to a medium containing 10 µg/ml of NB extract and incubated for a further 24 h.

### Western Blot Analysis

Colon cell lines were lysed in a RIPA lysis buffer-radioimmunoprecipitation (RIPA Buffer) (BioRad Laboratories Inc.), for 60 min at −20°C. The supernatant was collected after centrifugation at 13,200 rpm for 20 min. Protein concentration was determined spectrophotometrically using the Thermo Scientific Pierce Kit (BCA Protein assay kit) at 562 nm. Equal amounts of protein were prepared with 0.5 M Tris HCl at pH 6.8, 10% glycerol, 10% w/v SDS, 5% β2-mercaptoethanol. and 0.05% w/v bromophenol blue. A 30 μg/lane of protein and prestained ladder (Thermo Fisher Scientific, Waltham, MA, United States) were separated by SDS-PAGE and transferred to nitrocellulose membranes (Bio-Rad). Membranes were blocked with 5% nonfat milk in Tris-buffered saline containing 0.05% Tween 20 (TBST) at 4°C. Blots were incubated with primary antibodies at a dilution and time incubation according to the manufacturer’s protocol. The antibodies PARP Rabbit mAb Antibody #9532, ATF4 mAb Rabbit (D4B8) #1815, ATF-6 (D4Z8V) mAb Rabbit #65880, total-IRE1α (14C10) mAb Rabbit #3294, Phospho-JNK (Thr183/Tyr185) mAb Mouse antibody #9255, SAPK/JNK Antibody (total-JNK) pAb Rabbit #9252, CHOP (L63F7) mAb Mouse antibody #2895, BiP or GRP78 (C50B12) mAb Rabbit #3177, XBP-1s (D2C1F) mAb Rabbit #12782, Phospho-eIF2α (Ser51) (D9G8) mAb Rabbit #3398, total-eIF2α (D7D3) mAb Rabbit #5324, Caspase-3 Antibody #9662, and Caspase-9 #9502 were obtained from Cell Signaling Technology Inc., Beverly, MA, USA. Caspase-8 (human) monoclonal antibody (12F5) was obtained from Enzo Life Sciences, Inc., USA. The phospho-IRE1α antibody (S724) #ab124945 was obtained from Abcam, plc, United Kingdom. Anti-β-actin antibody was obtained from Sigma-Aldrich, St. Louis, MO, United States. After incubation with secondary antibodies for 1 to 3 h, bands of interest and densitometry were obtained with ChemiDoc^TM^ XRS + System (Bio-Rad) with Image Lab^TM^ software.

### Determination of Secondary Metabolites by HPLC-ESI-TOF-MS

The molecular composition of the dichloromethane:methanol extract from *Dolabella auricularia* obtained as described in Section *Preparation of Marine Extract* was characterized using High-Performance Liquid Chromatography coupled to Electrospray Ionization Quadrupole Time-of-Flight Mass Spectrometry (HPLC–ESI-Q-TOF-MS) in a previous study [13].

### Statistical Analysis

Data shown are the mean ± standard deviation (SD) of three independent assays. Statistical analysis was tested by one-way analysis of variance (ANOVA), followed by Tukey’s post hoc test for multiple comparisons using GraphPad Prism 7.02. Significant differences between experimental groups were assumed at *p*-values < 0.05 (*p-value < 0.05, **p-value < 0.01, ***p-value < 0.001, or ****p-value < 0.0001).

## Results

### NB Extract Inhibits the Proliferation of Adenocarcinoma Cell Lines and Is Less Cytotoxic in a Colon Fibroblast Cell Line

To investigate the cytotoxic effect of NB extract, HCT-116 and CCD-18Co cells were treated with various concentrations of NB extract for 24 h, and cell viability was assessed by the MTT assay. NB suppressed the proliferation of HCT-116 and normal CCD-18Co cells in a dose-dependent manner (Figures 1B,C). Importantly, the IC_50_ value of NB was 14.9-fold higher in normal CCD-18Co cells (15.04 ± 1.38 µg/ml) than in HCT-116 cells (1.01 ± 0.19 µg/ml); this comparison is commonly known as Selective Cytotoxicity Index (SCI) (Badisa et al., 2009). When SCI is equal or greater than 2, the treatment is considered as selective. The greater the SCI value of a compound is, the more selective it is. As the value for NB was 14.9, significantly greater than the limit of 2, the selective action of NB extract against colon cancer cells can be postulated.

Phase-contrast microscopy showed major alterations in cell morphology when HCT-116 cells were treated with 10 µg/ml NB (Figure 1D **)**. NB-treated cells have condensed nuclei, suggesting pyknosis, an expanded cytoplasm, membrane blebbing, and membrane protrusions, which are characteristic of apoptosis; untreated cells had normal morphology. On the contrary nontumor cells CCD-18Co did not show major changes in morphology.

The antiproliferative activity of NB was then characterized in HCT-116 cancer cells using the colony formation (clonogenic) assay and a real-time cell analyzer system (RTCA). The clonogenic assay provides information about the ability of cancer cells to survive and form a colony from a clone after a treatment (Pérez-Sánchez et al., 2018), and the RTCA assay monitors cellular processes such as proliferation, migration, and invasion in real time, avoiding labor-intensive label-based endpoint assays (Limame et al., 2012), which are translated to a CI. Treatment of HCT116 cells with NB extract resulted in a marked decrease in the number of colonies compared to untreated cells and reduced the average size of the colonies in a dose-dependent manner (Figures 1E,F **)**. NB extract at the lowest concentration of 0.5 μg/ml induced an 83.4% decrease in the size of colonies (CONT: 279.5 μm–NB: 46.5 μm) and a 49.8% reduction in the number of colonies (CONT: 81.3 cells–NB: 40.8 cells) compared with untreated cells. At higher concentrations of NB extract, a further reduction in the size and number of colonies was observed, leading to a minimum average size of 14.1 µm and a minimum average number of 4.5 cells at a concentration of 10 μg/ml.

Using the RTCA technique, the cell index of proliferation (CIP) of HCT-116 was registered as a kinetic cellular profile (Figure 1G). The kinetics of untreated cells showed an exponential phase until 60 h and a stable rate of growth until 72 h. When cells were treated with NB extract, the exponential phase was not observed, and the CIP was decreased at the lowest dose of 0.5 µg/ml NB extract after 8 h of treatment, at 1.0 and 10 µg/ml NB extract after 6 h of treatment, and at 25 µg/ml NB extract after 5 h of treatment. Collectively, these results indicate that NB extract inhibits the proliferation of HCT-116 cells in a dose-dependent manner.

### NB Extract Increases Intracellular ROS and Activates ER Stress–Related Proteins in HCT-116 Cells

**FIGURE 2:**
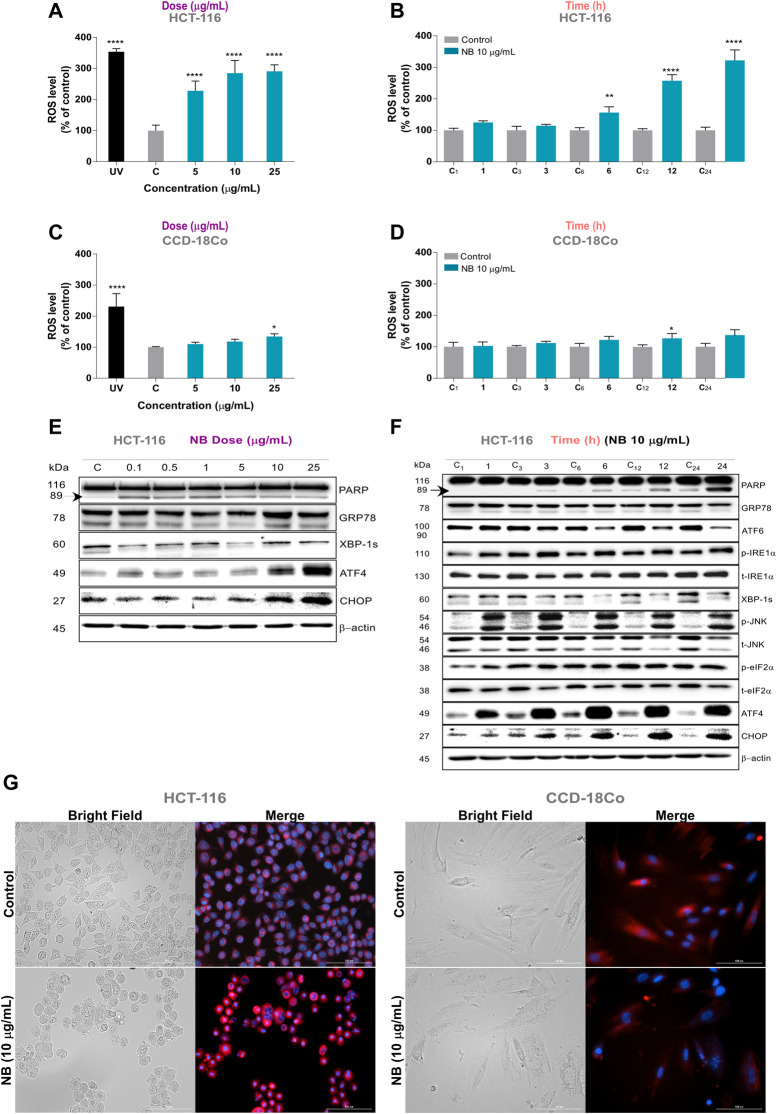
Effect of *Dolabella auricularia* extract (NB) on ROS generation and concomitant ER stress in colon cells. Intracellular ROS levels were measured in HCT-116 and CCD-18Co using the 2′,7′-dichlorodihydrofluorescein diacetate (H_2_DCF-DA) fluorescence probe after NB treatment in a dose- **(A**,**C)** and time-dependent manner **(B**,**D)**. Time in hours is indicated on the X axis and is preceded by “C” for control samples. Data were compared to the control (untreated cells plus less than 0.2 % DMSO) and are presented as the mean ± SD of three independent experiment. *p*-values were calculated by comparison with untreated cell lines using ANOVA. *p-value < 0.05, **p-value < 0.01, *** *p*-value < 0.001, and **** *p*-value < 0.0001. The ability of NB extract to modulate ER stress-related proteins in HCT-116 cells was analyzed by western blot analysis. Effect of different concentration of NB after 24 h incubation is shown in **E** and effect of 10 µg/mL of NB in time is shown in **F**. Time (h) is indicated on the upper part and is preceded by “C” for control samples. β-Actin was used as a loading control. Densitometry analyses of **E** and **F** are shown in Supplementary Figures 1 and 2, respectively. Representative images of HCT-116 (left) and CCD-18Co (right) cells treated with 10 µg/mL NB extract in bright field and merged fluorescence channels corresponding to DAPI (Hoechst, nucleus) and Texas red (ER Tracker Red, endoplasmic reticulum) at 20 × (**G**).

Treatment of HCT-116 cells with NB extract resulted in a significant increase in intracellular ROS levels in a dose- and time-dependent manner (Figures 2A,B). The level of ROS increased up to 2.3-fold at 5 µg/ml NB extract (CONT: 100 ± 17.9% - NB5: 228.4 ± 31.0%). On the contrary, only a minor increase in the ROS level was observed in normal CCD-18Co cells after treatment with NB extract at the highest concentration of 25 µg/ml (Figures 2C,D).

ROS are generated after exposure to physical and chemical agents and are produced in mitochondria (*via* the electron transport chain), peroxisomes (*via* β-oxidation of fatty acids), and the ER (*via* protein oxidation). ROS are closely linked to the activation of the unfolded protein response (UPR) and ER stress, leading to apoptosis, and play an important role in the downregulation of cell cycle progression and cell survival pathways (Banerjee et al., 2017; Perillo et al., 2020). To determine whether activation of the UPR pathways is related to the antiproliferative effect of NB extract, the expression and activation of several proteins involved in the 3 UPR pathways were evaluated by western blot analysis after the treatment with NB extract (dose-dependent, Figure 2E time-dependent Figure 2F); the densitometry analysis results are shown in Supplementary Figures S1, S2.

Treatment of HCT116 cells with 10 and 25 µg/ml NB extract resulted in acute upregulation of ATF4 and CHOP (Figure 2E). Based on these results, 10 µg/ml of NB was considered the effective concentration selected for further ER stress assays. The changes in the UPR protein levels over time after treatment with 10 µg/ml NB extract are shown in Figure 2F. The data indicate that the phospho-JNK, phospho-eiF2α, ATF4, and CHOP levels are upregulated as early as 3 h after treatment with NB extract. A slight induction in cell death, as shown by the increase in cleaved PARP, was shown at 6 h after treatment with NB extract, which became more significant after 24 h.

When ER stress is produced, mammalian cells try to cope with the folding load by massively expanding the ER membrane. Figure 2G (left) shows that treatment of HCT-116 cells with 10 µg/ml NB for 24 h dramatically increased the size of the ER, with higher fluorescent intensity, as determined using the ER-Tracker probe. Conversely, the nontumoral cell line showed no significant increase in the fluorescent signal, and nor any plausible increase in the volume of the ER (Figure 2G, right).

### NB Extract Induces Strong G2/M Cell Cycle Phase Arrest and Cell Death as a Consequence of ER Stress in Colon Cancer Cell Lines

**FIGURE 3:**
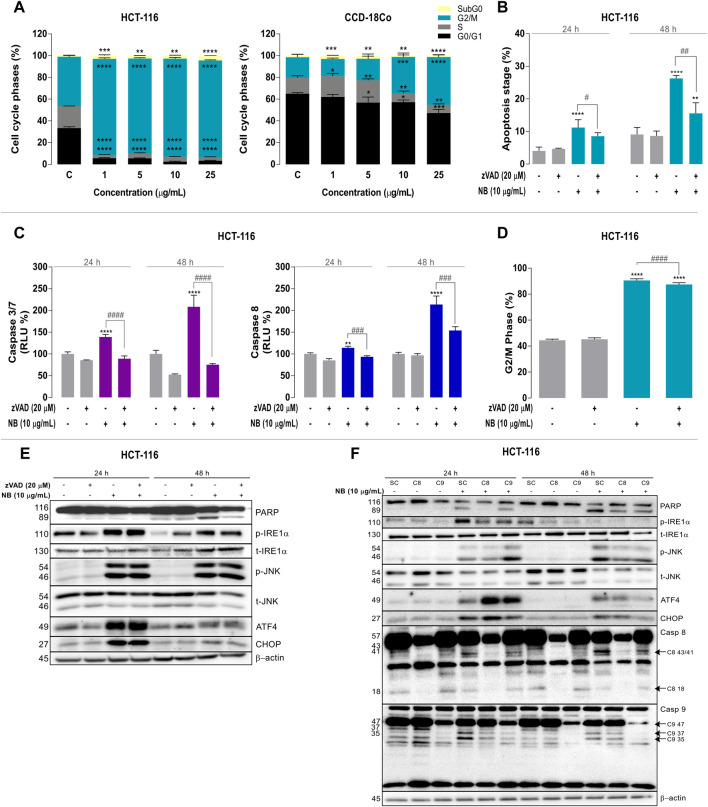
Relationship among ER stress, cell cycle modulation, and caspase-dependent apoptotic cell death in colon cancer cells treated with *Dolabella auricularia* extract (NB). **(A)** Effect of increasing concentrations of NB extract on the cell cycle of HCT-116 and CCD-18Co cells after 24 h. HCT-116 cells were pretreated with a pan-caspase inhibitor, zVAD (20 μM), for 2 h prior to the addition of 10 μg/ml NB extract for 24 h to study the effect of NB extract on apoptosis. **(B)** Measurement of apoptotic cells (% Annexin V positive cells), **(C)** caspases 3/7 and 8, and **(D)** the G2/M phase. Data are presented as the mean ± SD of three independent experiments. *p*-values were calculated by comparison with the untreated cell line (*) and NB condition (**#**) using ANOVA. * or ^#^ *p*-value < 0.05, ** or ^##^ *p*-value < 0.01, *** or ^###^ *p*-value < 0.001, and ****or ^####^ *p*-value < 0.0001. The effects of NB extract on the modulation of ER stress–related proteins and apoptosis were analyzed by western blot analysis. The effect of NB extract on ER stress–related proteins **(E)** and its relationship to caspase activation **(F)**. Caspases 8 and 9 in HCT-116 were silenced with siRNA, and cells were treated with 10 μg/ml NB extract for 24 h. ER stress–related proteins (PARP, phospho-IRE1α, total-IRE1α, phospho-JNK, total-JNK, ATF4, CHOP, and caspases 8 and 9) were analyzed by western blot analysis. β-Actin was used as a loading control in the western blot assay. Densitometry analyses of **E** and **F** are shown in Supplementary Figures 3 and 4, respectively.

The observed antiproliferative effects of NB extract on the colon cancer cells and concomitant ER stress were further explored by evaluating the effects of NB extract on the cell cycle in the HCT-116 cells and the normal colon cell line CCD-18Co. Treatment with NB extract resulted in a significant arrest of tumor cells in the G2/M phase. As shown in Figure3A left, NB extract at the lowest dose of 1.0 µg/ml induced a dramatic 2.0-fold increase in the fraction of HCT-116 tumor cells in the G2/M phase (CONT: 45.05 ± 1.33%-NB: 89.12 ± 0.52%). In CCD-18Co cells, this effect was significantly lower (Figure 3A right), i.e., the fraction of cells in the G2/M phase was enhanced by 1.3-fold (CONT: 18.91 ± 2.72% - NB: 33.49 ± 3.68%) at 10 µg/ml NB and 2.3-fold at 25 µg/ml NB (CONT: 18.91 ± 2.72%-NB: 43.67 ± 1.99%).

Furthermore, the 25 µg/ml NB extract treatment increased the proportion of HCT-116 cells in the subG0 state by 4.8-fold (CONT: 0.9 ± 0.2%–NB: 4.35 ± 0.12%).

#### Association of ER Stress and G2/M Cell Cycle Arrest Induced by NB Extract with Caspase-Dependent Apoptosis in Colon Cancer Cells

We then investigated whether the ER stress induced by NB and the subsequent cell cycle arrest were related to caspase-dependent apoptosis in HCT-116 CRC cells using a cell-permeable caspase inhibitor that blocks the activity of caspase proteases and inhibits apoptosis, Z-VAD-FMK (zVAD).

The mechanism of cell death after the 10 μg/ml NB extract treatment was examined using the Annexin V/7-ADD apoptosis assay (Figure 3B). The level of apoptosis (Annexin V positive cells percentage) was increased by 2.7-fold (CONT: 4.1 ± 1.1% - NB: 11.2 ± 2.4%) at 24 h and by 2.9-fold (CONT: 9.1 ± 2.2%-NB: 26.3 ± 1.0%) at 48 h. When caspases were inhibited with zVAD, the fraction of Annexin V positive cells was reduced by 0.8-fold at 24 h (NB10: 11.2 ± 2.4%-NB10 + zVAD: 8.6 ± 1.0%) and by 0.6-fold at 48 h (NB10: 26.3 ± 0.9%-NB10 + zVAD: 15.6 ± 3.2%).

Caspases 3/7 and 8 were particularly involved in the induction of apoptosis after the 10 μg/ml NB treatment (Figure 3C) in HCT-116 cells. NB treatment for 24 h resulted in a significant 1.4-fold increase in the activity of the executioner C3/7 caspase (CONT: 100.0 ± 4.8%-NB: 139.3 ± 5.7%), and its activity was significantly decreased by 0.6-fold after cotreatment with zVAD (NB: 139.3 ± 5.7% - NB + zVAD: 89.2%). A similar pattern at a greater scale was observed at 48 h (Figure 3C left). Treatment with 10 µg/ml NB extract increased C8 activity by 1.1-fold, which was suppressed by 0.8-fold after cotreatment with zVAD at 24 h (CONT: 100.0 ± 2.7% - NB: 114.0 ± 4.0% - NB + zVAD: 93.6 ± 2.5%). Similarly, C8 activity was increased by 2.1-fold after 48 h treatment with NB extract, and this effect was significantly decreased by 0.7-fold by cotreatment with zVAD (CONT: 100.0 ± 3.9% - NB: 213.5 ± 19.7%-NB + zVAD: 154.2 ± 8.4%) (Figure 3C right).

The ability of NB extract to increase the percentage of cells in the G2/M phase is one of its most remarkable effects. Figure 3D shows the effect of NB extract on the percentage of cells arrested in the G2/M phase after 24 h and its possible relationship with the activation of apoptosis in the presence of zVAD. The G2/M arrest after 24 h treatment with NB extract was slightly reduced by inhibiting caspases with zVAD (CONT: 44.5 ± 0.9%-NB: 90.6 ± 1.2%-NB + zVAD: 87.4 ± 1.4%).

The relationship between apoptosis and the activation of ER stress markers was analyzed by western blot analysis (Figure 3E; densitometry is shown in Supplementary Figure S3). When caspases were inhibited, cell death (cleaved PARP) after NB treatment was decreased, indicating the dependence of the cell death processes on caspases. Pronounced activation of ER stress proteins, such as phospho-IRE1α, phospho-JNK (at 24 and 48 h), and the nuclear transcription factor ATF4, resulted in their persistent upregulation despite the inhibition of caspases; only the level of CHOP was reduced at 48 h. These results indicate that NB-induced UPR activation was not the result of caspase activation.

To define the relative importance of the extrinsic and intrinsic apoptotic pathways in the mediation of NB extract–induced apoptosis, we used siRNAs specifically directed against caspase-8 (extrinsic pathway) or caspase-9 (intrinsic pathway) (Figure 3F; Supplementary Figure S4). Treatment with NB extract resulted in marked cleavage of caspase 8 (p43/41) (Supplementary Figure S4F) and caspase 9 (p37/35) (Supplementary Figure S4G **)**. To study the implication of caspases in NB apoptotic cell death, we compared cleavage of caspases 8 and 9 with cleavage of PARP. NB extract treatment resulted in marked cleavage of the apoptotic cell death marker (PARP). It was increased at 24 and 48 h, which was reduced by caspase-8 silencing at 24 h with statistical significance. Similar to the results obtained with zVAD, siC8 did not influence the NB-dependent increase in CHOP, ATF4, or phospho-JNK expression. Although caspase 9 cleavage was activated by NB, its silencing is not related to a reduction in PARP cleavage. This result suggests that cell death induced by NB extract proceeds via caspase-8-mediated activation of the caspase-9-dependent intrinsic apoptotic pathway.

#### The Effect of NB Extract Treatment of Cells on Caspase-Independent Cell Death

Previous results (Section *Association of ER Stress and G2/M Cell Cycle Arrest Induced by NB Extract with Caspase-dependent Apoptosis in Colon Cancer Cells*) showed that activation of early apoptosis (phosphatidylserine (PhS) externalization) and PARP cleavage were induced after NB extract treatment. When caspases were inhibited using zVAD, these markers were reduced; however, the activation remained, indicating additional putative caspase-independent cell death. Therefore, the potential presence of other alternative cell death mechanisms, such as necrosis, necroptosis, or autophagy, was analyzed in HCT-116 cells after NB extract treatment for 24 h. The results shown in Supplementary Figure S5 discarded necrosis, necroptosis, or autophagy as the putative cell death mechanisms of NB extract in the colon cancer HCT-116 cell model.

### Is ER Stress the Cause or the Consequence of the NB Effect on ROS?

NB extract induces ROS and the concomitant activation of some ER stress proteins in the HCT-116 CRC cells. ROS and ER stress are intricately connected (Ozgur et al., 2014; Banerjee et al., 2017); to clarify whether ROS are the upstream trigger of the NB effect, a ROS scavenger, N-acetyl cysteine (NAC, 5 mM), and an ER stress inhibitor, 4-phenylbutyric acid (4-PBA, 5 mM (Sarvani et al., 2017)), were added to cells 2 h before treatment with NB extract (10 μg/ml).

**FIGURE 4:**
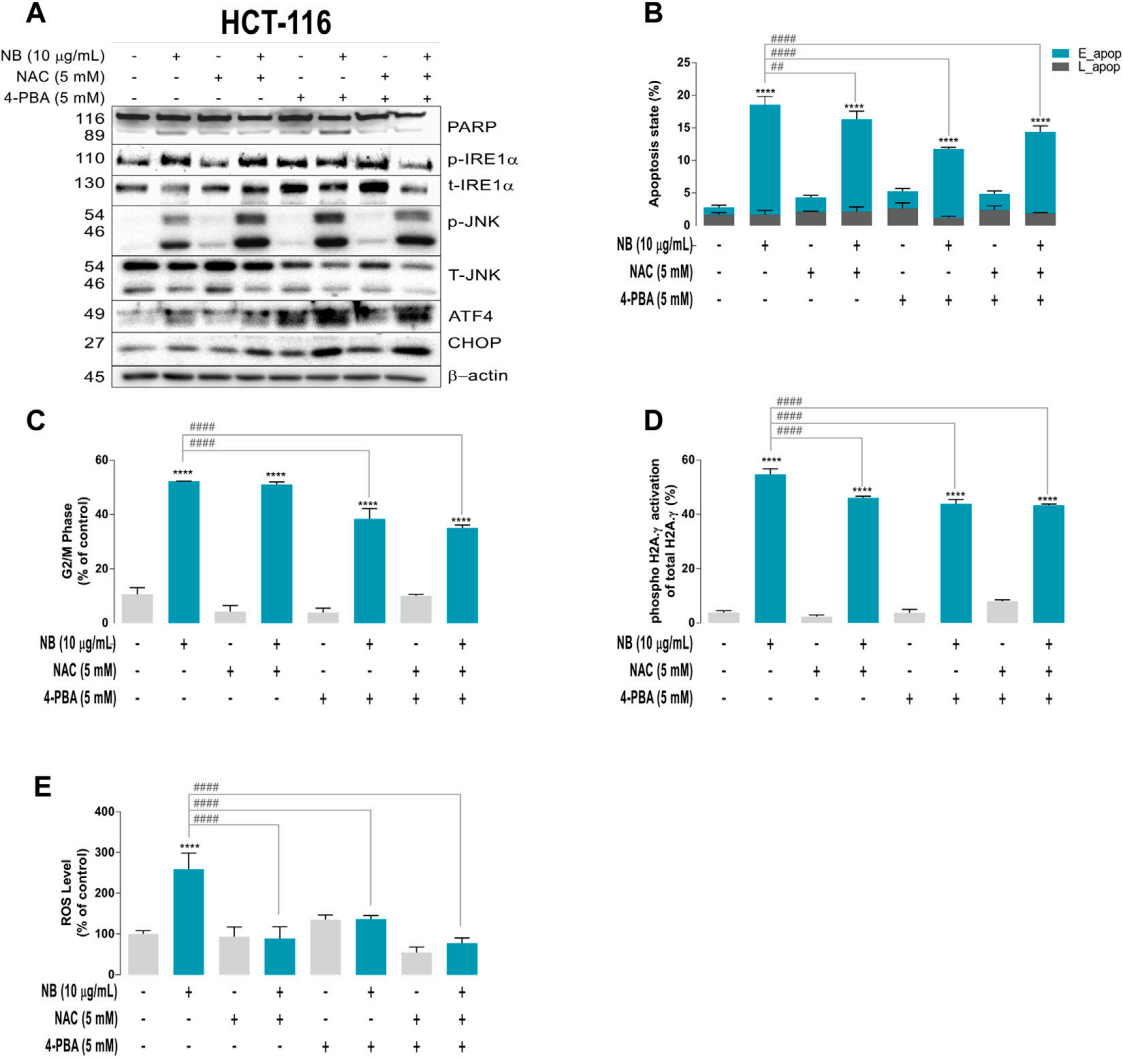
Relationships between ROS and ER stress induced by NB extract in the colon cancer HCT-116 cell model. Cells were pretreated with the ROS scavenger N-acetyl-cysteine (NAC, 5 mM) and ER stress inhibitor 4-phenylbutric acid (4-PBA, 5 mM) for 2 h prior to 10 μg/ml NB extract treatment. NB extract modulation of ER stress–related proteins (PARP, phospho-IRE1α, total-IRE1α, phospho-JNK, total-JNK, ATF4, and CHOP) was analyzed by western blot analysis **(A)**. β-Actin was used as a loading control. The experiment was repeated twice. Apoptosis **(B)**, G2/M phase **(C),** and phosphorylation of H2A.γ (as DNA damage indicator) **(E)** were measured using a Muse^®^ cell analyzer according to the manufacturer’s instructions. ROS levels were measured using the 2′,7′-dichlorodihydrofluorescein diacetate (H_2_DCF-DA) fluorescence label **(D)**. Data are presented as the mean ± SD of three independent experiments. *p*-values were calculated by comparison with the control (untreated cells plus less than 0.2% dimethyl sulfoxide, C) (*) and NB condition (**#**) using ANOVA. * or ^#^ *p*-value < 0.05, ** ^or ##^ *p*-value < 0.01, *** or ^###^ *p*-value < 0.001, and **** ^or ####^ *p*-value < 0.0001.

Figure 4A (densitometric analysis is shown in Supplementary Figure S6) illustrates the expression of ER stress–related proteins after NB extract treatment of HCT-116 cells that were pretreated with ROS or ER stress inhibitors or their combination. In the CRC cell line HCT-116, the ROS scavenger NAC was the only inhibitor that was able to reduce cell death (lower PARP cleavage). NAC also reduced the expression of the transcription factors ATF4 and CHOP, suggesting that ROS are directly related to these ER stress markers. Although 4-PBA and its combination with NAC did not reduce cell death (the cleavage of PARP was not reduced), these compounds inhibited phospho-IRE1α. These results indicate that ROS are the main inducers of ER stress in the colon cancer cell line (Figure 4A and the densitometric analysis shown in Supplementary Figure S6), and this effect was not observed in the normal colon cell line CCD-18Co (Supplementary Figures S7, S8) since NB did not promote the cleavage of PARP (cell death) or activation of ER stress–related proteins.

Figure 4B shows the contribution of inhibition of ROS and/or ER stress on the induction of apoptosis in HCT-116 cells treated with NB extract. The results show that both inhibitors slightly reduced early apoptosis. However, NB did not induce a significant apoptotic effect in the normal cell line CCD-18Co (Supplementary Figure S7B).

Figure 4C shows the contribution of ER stress and ROS to the G2/M arrest of cells induced by NB. As shown in previous sections, 10 µg/ml NB extract treatment for 24 h induced G2/M cell cycle arrest of HCT-116 cells (CONT: 44.3% vs. NB: 90.2%), which was significantly reduced by 4-PBA and its combination with NAC (4-PBA: 80.4%). On the other hand, NB did not have the same effect in the normal colon cell line. The G2/M cell cycle arrest of CCD-18Co cells after NB extract treatment increased slightly (CONT: 20.1% vs. NB: 26.6%), and NAC, 4-PBA, and their combination did not significantly reduce the effect of NB extract (Supplementary Figure S7C).

Figure 4D shows the effect of both inhibitors on ROS generation. NB extract induced a 2.6-fold increase in ROS (CONT: 100 ± 8.4% - NB: 259.2 ± 39.4%) in HCT-116 cells. Treatment with the ROS scavenger NAC reduced ROS by 0.3-fold, 4-PBA reduced ROS to a lesser degree (0.5-fold), and the combination of NAC and 4-PBA induced the highest ROS decrease of 0.3-fold, indicating the induction of both types of stress after treatment with NB extract (NB: 259.2 ± 39.4%-NB + NAC: 89.2 ± 28.6%-NB+4-PBA: 136.1 ± 9.5%-NB + NAC+4PBA: 77.8 ± 12.6%). In contrast, in the normal cell line CCD-18Co, the ROS increase induced by 10 µg/ml NB extract was less pronounced than that in HCT-116 cells and was reduced by NAC, 4-PBA, and their combination (Supplementary Figure S7D).

Figure 4E shows the activation of H2A.γ as an indicator of DNA damage caused by NB extract in HCT-116 and CCD-18Co cells. NB extract induced a 14.4-fold activation of H2A.γ in HCT-116 cells, which was reduced by NAC (0.8-fold), 4-PBA (0.8. fold), and their combination (0.8-fold; NB: 54.7 ± 2.0%-NB + NAC: 46.1 ± 0.6%-NB+4-PBA: 43.8 ± 1.6%-NB + NAC+4PBA: 43.3 ± 0.5%; the differences were statistically significant). In the normal colon cell line CCD-18Co, NB extract induced activation of H2A.γ (CONT: 1.2 ± 0.7–NB: 23.2 ± 0.8%), which was drastically reduced by NAC and slightly reduced by 4-PBA and the combination of both inhibitors (NB: 23.2 ± 0.8% - NB + NAC: 1.9 ± 0.8% - NB+4-PBA: 19.6 ± 1.4%–NB + NAC+4PBA: 17.0 ± 0.8%) (Supplementary Figure S7E).

### NB Extract Suppresses the Proliferation, Migration, and Invasion of Parental HCT-116 Cells and Invasive Populations

**FIGURE 5:**
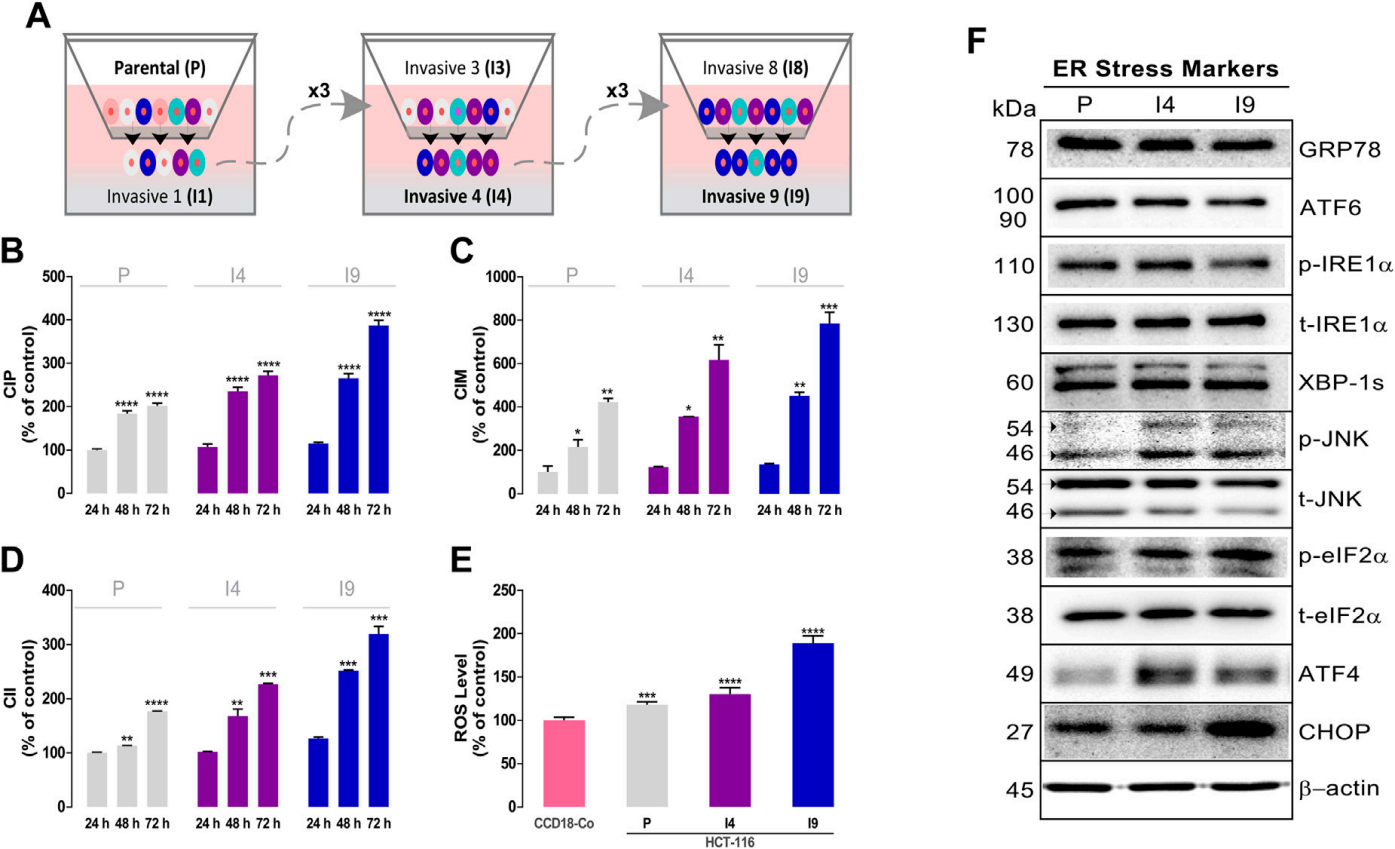
Isolation and characterization of superinvasive populations of HCT-116 cells. **(A)** Schematic representation of the isolation process of the invasive 4 (I4) and invasive 9 (I9) cell lines. A Real-Time Cell Analyzer (RTCA) dual-plate instrument (Roche Diagnostics GmbH, Germany) was used to measure the basal proliferation cell index (CIP) using an E-plate **(B)**, migration cell index (CIM) using a CIM-plate **(C)**, and invasion cell index (CII) using a CIM-plate coated with Matrigel **(D)** of the superinvasive populations of HCT-116. **(E)** ROS levels were measured using the 2′,7′-dichlorodihydrofluorescein diacetate (H_2_DCF-DA) fluorescence label. Data are presented as the mean ± SD of three independent experiments. *p*-values were calculated by comparison with the control (untreated cells plus less than 0.2% dimethyl sulfoxide, C) using ANOVA. *p-value < 0.05, ** *p*-value < 0.01, *** *p*-value < 0.001, and **** *p*-valu e< 0.0001. **(F)** The basal levels of ER stress–related proteins in superinvasive HCT-116 populations were assayed using western blot analysis. β-Actin was used as a loading control. The experiment was repeated twice. (Densitometric analysis is shown in Supplementary Figure S9)

To test the effect of NB effect on differentiated malignant cells, highly invasive populations of HCT-116 cells were obtained. Isolation of these cells was performed by serial selection using Boyden chambers starting with the parental (P) cell line and was repeated to obtain the invasive 4 population (I4) and superinvasive I9 population (I9) (Figure 5A).

Initially, the proliferation (Figure 5B), migration (Figure 5C), and invasion rate (Figure 5D) of the parental and invasive HCT-116 subpopulations were assessed using the RTCA technique. The data showed that the I4 and I9 invasive populations had the highest cell index of proliferation (CIP) and higher migration (CIM) and invasiveness (CII) than the parental population. At 48 h, I4 showed 1.3-fold and I9 showed 1.4-fold higher average CIP values than P (P _48 H_: 183.4 ± 6.8%-I4 _48 H_: 235.3 ± 9.3%-I9 _48 H_: 265.4 ± 10.8%). I4 had 1.6-fold and I9 had 2.1-fold higher CIM values than P (P _48 H_: 216.1 ± 32.7%-I4 _48 H_: 355.6 ± 0.9% - I9 _48 H_: 451.5 ± 16.6%). The same pattern was detected in CII. I4 had 1.5-fold and I9 had 2.2-fold higher CII values than *p* (P _48 H_: 113.7 ± 0.2%-I4 _48 H_: 167.8 ± 13.0%-I9 _48 H_: 252.0 ± 1.3%).

The basal ROS levels of the highly invasive I4 and I9 HCT-116 subpopulations, P, and nontumor CCD-18Co cells were also measured. Figure 5E shows that the highest basal ROS levels were detected in the I9 invasive cell line, followed by I4 and P of HCT-116 cells.

To determine the effect of NB on ER stress–related proteins in the invasive HCT-116 cell subpopulations, we assessed the basal levels of ER stress–related proteins (images in Figure 5F and densitometry in Supplementary Figure S9). The results indicate that the phospho-JNK and ATF4 levels were markedly increased in I4 and I9 invasive HCT116 cells compared with P cells, and the CHOP levels were mainly upregulated in the I9 subpopulation.

**FIGURE 6:**
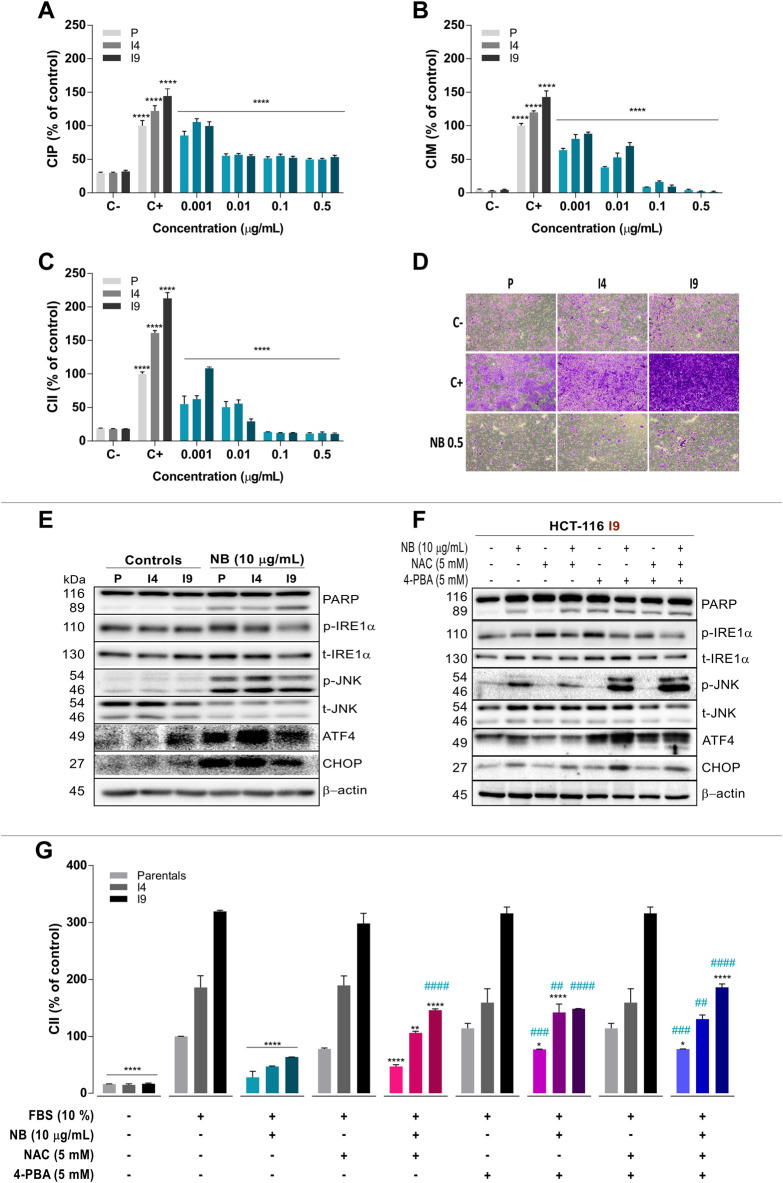
Effect of NB on superinvasive populations of HCT-116 cell lines. The P, I4, and I9 cell lines were seeded on an E-Plate to assay the cell index of proliferation (CIP) **(A)**, on a CIM-Plate to assay the cell index of migration (CIM) **(B),** and on a CIM-Plate coated with Matrigel to assay the cell index of invasion (CII) **(C)**; the changes induced by NB treatment were measured. Data are presented as the mean ± SD of three independent experiments. Cell index data from the negative control (untreated cells in FBS-free media, C-) were compared to those from the positive controls (untreated cells cultured in media with 10% FBS, C+). The cell index of HCT-116 P, I4, and I9 cells treated with NB extract was compared to C+. Data are presented as the mean ± SD of three independent experiments. *p*-values were calculated by comparison with the untreated cell line controls (control cells plus less than 0.2% dimethyl sulfoxide, (C) using ANOVA. *p-value < 0.05, ** *p*-value < 0.01, *** *p*-value < 0.001, and **** *p*-value < 0.0001. **D)** Representative images (4X) of the P, I4, and I9 invasive populations stained with crystal violet in a transwell insert are shown. Untreated cells were used as a negative control (C-, 0% FBS, 0% invasion) and positive control (C+, 10% FBS, 100% invasion) and were compared to NB extract-treated superinvasive cells (0.5 μg/ml NB) for 48 h. **(E)** The effect of NB extract on ER stress–related proteins from the superinvasive populations was analyzed by western blot analysis. β-Actin was used as a loading control. The experiment was repeated twice. (Densitometric analysis is shown in Supplementary Figure S10). **(F)** The relationship between ROS and ER stress induced by NB extract in the superinvasive HCT-116 I9 subpopulation was analyzed. The I9 cell line was pretreated with NAC (5 mM), 4-PBA (5 mM), and their combination for 2 h prior to the 10 μg/ml NB treatment, and ER stress markers were assayed by western blot. β-Actin was used as a loading control. The experiment was repeated twice. (Densitometric analysis is shown in Supplementary Figure S11). **(G)** The P, I4, and I9 cell lines were pretreated with NAC (5 mM), 4-PBA (5 mM), and their combination for 2 h prior to the 10 μg/ml NB extract treatment. The CII was measured by the RTCA system. The cell index was compared to the negative control (untreated cells in FBS-free media, 0% invasion) and positive control (untreated cells cultured in media with 10% FBS, 100% invasion). The experiment was repeated twice. The data are presented as the mean ± SD. *p*-values were calculated by comparison to the same untreated population (*) or to the same population treated with NB (#) using ANOVA. *p-value < 0.05, **p-value < 0.01, ***p-value < 0.001, and ****p-value < 0.0001 or ^#^ *p*-value < 0.05, ^##^ *p*-value < 0.01, ^###^ *p*-value < 0.001, and ^####^ *p*-value < 0.0001.

The effect of NB extract on the proliferation, migration, and invasion of the invasive HCT-116 subpopulations is shown in Figure 6A. The increased proliferation (CIP) of the superinvasive I4 and I9 populations was slightly reduced after NB extract treatment. At the lowest tested concentration (0.001 µg/ml NB extract), the CIP levels were decreased by 0.9-fold in *p* (*p*_C+_: 100.0 ± 7.8%-P _NB_: 85.6 ± 6.1%), 0.4-fold in I4 (I4 _C+_: 122.5 ± 7.7%-I4 _NB_: 105.7 ± 4.9%), and 0.7-fold in I9 (I9 _C+_: 144.5 ± 10.8%-I9 _NB_: 99.7 ± 6.3%). More significant reductions were obtained at higher concentrations of NB extract.

NB extract was able to lessen the CIM of the invasive I4 and I9 HCT-116 populations (Figure 6B). At the lowest tested concentration of NB extract (0.001 µg/ml), the CIM of P was reduced by 0.6-fold (P _C+_: 100.0 ± 3.6% - *p*_NB_: 63.6 ± 2.7%), CIM of I4 was reduced by 0.7-fold (I4 _C+_: 120.1 ± 2.1%-I4 _NB_: 80.5 ± 6.5%), and CIM of I9 was reduced by 0.6-fold (I9 _C+_: 143.0 ± 9.2%-I9 _NB_: 88.2 ± 2.6%). At higher concentrations of NB (0.01, 0.1, and 0.5 µg/ml) the effect of NB extract on CIM was intensified and reached values similar to those in the negative control (0% migration).

The invasive cell index (CII) was also reduced after NB extract treatment (Figure 6C **)** at the lowest tested concentration. At 0.001 µg/ml NB extract, the CII of P was decreased by 0.6-fold (*p*_C+_: 100.0 ± 3.1%-P _NB_: 55.0 ± 11.9%), CII of I4 was decreased by 0.4-fold (I4 _C+_: 161.1 ± 3.8%-I4 _NB_: 62.7 ± 5.0%), and CII of I9 was decreased by 0.5-fold (I9 _C+_: 212.8 ± 8.8%-I9 _NB_: 108.7 ± 1.9%). At higher concentrations of NB extract (0.01, 0.1, and 0.5 µg/ml), the inhibition of CII was increased, reaching the values of the negative control (0% invasion). Figure 6D shows microphotographs of invasive HCT-116 P, I4, and I9 cells labeled with crystal violet of the negative control (0% invasion), positive control (100% invasion), and after NB extract treatment samples. At 0.5 µg/ml NB extract, the inhibition of invasive cells was nearly complete, reaching the values obtained in the negative control.

The next experiment aimed to verify whether NB extract had the same effect on ER stress–related proteins in the invasive sublines I4 and I9 as it did in the *p* line. Treatment with 10 µg/ml NB extract for 24 h resulted in acute upregulation of phospho-JNK, ATF4, and CHOP in the parental line and invasive sublines (images in Figure 6E and densitometry data in Supplementary Figure S10), which was associated with an increase in cell death (PARP cleavage).

Our data indicate that NB extract is a potent activator of oxidative stress and ER stress in CRC HCT-116 cells and a strong inhibitor of proliferation, migration, and invasion of HCT-116 populations. To determine whether the ER stress and ROS induced by NB extract are directly responsible for the decreased invasion observed in cells treated with NB extract, an inhibitor of ER stress, 4-PBA, and a ROS scavenger, NAC, and their combination were used. ER stress markers and cell death in I9 (the most invasive subline) were assayed by western blot analysis (images in Figure 6F and densitometry data in Supplementary Figure S11). Similar to the previous results, 10 µg/ml NB extract treatment for 24 h activated the I9 ER stress–related proteins phospho-JNK, ATF4, and CHOP. Only pretreatment with NAC, not with the other inhibitors, abrogated the NB extract–induced ERS-activated protein levels and markedly reduced cell death (PARP cleavage) **(** Figure 6F).

Furthermore, the effect of the inhibitor of ROS and 4-PBA on the NB extract-dependent inhibition of the invasiveness (CII) of the invasive populations was also studied **(** Figure 6G). Treatment with 10 µg/ml NB extract substantially reduced the CII in P, I4, and I9 cells. Reduction of ROS by NAC recovered the CII by 18.6% in P (P _C+_: 100.0 ± 0.3%-P _NB_: 28.6 ± 10.5%-P _NB+NAC_: 46.9 ± 3.4%), 59.3% in I4 (I4 _C+_: 185.8 ± 20.7%-I4 _NB_: 46.9 ± 1.1%-I4 _NB+NAC_: 106.2 ± 2.8%), and 82.4% in I9 (I9 _C+_: 319.2 ± 2.0%-I9 _NB_: 63.5 ± 0.3%-I9 _NB+NAC_: 145.9 ± 2.3%). When ER stress was inhibited by 4-PBA, invasion was recovered by 48.9% in P (P _C+_: 100.0 ± 0.3%-P _NB_: 28.6 ± 10.5%-P _NB+4PBA_: 77.2 ± 0.3%), 95.2% in I4 (I4 _C+_: 185.8 ± 20.7%-I4 _NB_: 46.9 ± 1.1%-I4 _NB+4PBA_: 142.1 ± 14.5%), and 85.0% in I9 (I9 _C+_: 319.2 ± 2.0%-I9 _NB_: 63.5 ± 0.3%-I9 _NB+4PBA_: 148.5 ± 0.3%). The combination of NAC and 4-PBA reversed the inhibitory effect of NB extract on invasion by 49.3% in *p* (*p*_C+_: 100.0 ± 0.3% - *p*_NB_: 28.6 ± 10.5% - *p*_NB+NAC+4PBA_: 77.6 ± 0.3%), 83.6% in I4 (I4 _C+_: 185.8 ± 20.7%-I4 _NB_: 46.9 ± 1.1%-I4 _NB+NAC+4PBA_: 130.5 ± 7.1%), and 122.6% in I9 (I9 _C+_: 319.2 ± 2.0%-I9 _NB_: 63.5 ± 0.3%-I9 _NB+NAC+4PBA_: 186.2 ± 6.0%).

## Discussion

The results of this work demonstrate that the extract (NB) from the marine nudibranch *Dolabella auricularia* has antiproliferative effects on HCT-116 CRC cells (Ruiz-Torres et al., 2019), and this effect is selective in contrast to that on normal colon cell lines. Compared to HCT-116 CRC cells, the NB extract–induced cytotoxicity was lower in the normal colon cell line CCD-18Co under similar conditions. These results suggest the existence of a specific and differential metabolic context, which differentiates tumor cells from normal colon cells and makes them more sensitive to marine NB extract. Nudibranchs are known to accumulate metabolites in their skin to protect them from predators (Cimino et al., 1985). Pyropheophorbide A, a chlorophyll-related compound, is one of the main compounds in *Dolabella auricularia* extract (Ruiz-Torres et al., 2019). This compound was initially identified in the edible red seaweed *Grateloupia elliptica* and was shown to have strong anticancer activity in U87MG glioblastoma cells mediated by cell cycle arrest, late apoptosis, and DNA degradation (Cho et al., 2014). Some of the major compounds of NB extract have not been studied in cancer. These compounds are terpenes, fatty acids, and steroids; many of these types of compounds from marine sources have attracted considerable research attention due to their potential antitumor activity (Ruiz-Torres et al., 2017).

When cells were treated with NB extract, an increase in ROS was observed concomitant to an increase in ER volume and activation of early ER stress markers after a short time of exposure, indicating that these processes play an important role in the antiproliferative effect. Various cell alterations can disturb protein homeostasis, leading to the aggregation of unfolded proteins in the ER (Scriven et al., 2007). When cells detect the accumulation of toxic unfolded proteins within the ER lumen, they try to adapt and restore cell homeostasis in a survival reaction known as the unfolded protein response (UPR) (Gardner and Walter, 2011). The UPR includes an increase in chaperones (such as GRP78); activation of stress sensors to limit the overload of unfolded proteins, such as inositol-requiring enzyme 1α (IRE1α), protein kinase RNA (PKR)-like kinase (PERK), and the transcription factor 6 (ATF6); reduction in protein transit through the ER; and an increase in the ER volume to stimulate membrane lipids, leading to endoplasmic reticulum-associated protein degradation (ERAD) (Gardner and Walter, 2011). After chronic ER stress, IRE1α, PERK, and ATF6 promote the activation of transcription factors to activate the cell death machinery, leading to apoptosis, autophagy, or necrosis (Sano and Reed, 2013; Kang et al., 2014; Banerjee et al., 2016). The results of the present study indicate that the first ER stress–related protein activated by NB extract is phospho-JNK, followed by early activation of ATF4 3 h after treatment with NB extract; then, CHOP is activated after 6 h, and finally, PARP cleavage and cell death occur at 24 h. The IRE1-α is an ER-transmembrane sensor that is activated by ER stress with endoribonuclease activity, and it activates mature XBP-1, a transcription factor that promotes the expression of several genes involved in the UPR and ERAD and regulates the expression the genes, such as CHOP (Kim et al., 2008). On the other hand, IRE1-α stimulates the activation of apoptosis signal-regulating kinase 1 (ASK1), which interacts with TNF receptor-associated factor 2 (TRAF2), activating c-Jun amino-terminal kinase (JNK), and p38 mitogen-activated protein kinase (p38 MAPK), which also induce CHOP and apoptosis (Hu et al., 2018). Thus, NB extract induces a significant increase of ROS after 6 h concurrent with CHOP activation (after an increase in the expression of phospho-JNK and ATF4) in CRC HCT-116 cells. ROS are known to mediate ER stress via JNK/p38 activation and induce apoptosis in several human cancer cells (Lin et al., 2018). JNK kinases are a family of proteins that regulate the stress signaling pathways related to gene expression, cell death, and the regulation of cellular senescence (Yarza et al., 2015). ROS can activate various kinases, including JNK, through TNFα. The activation of JNK can be qualitatively different, early and transient or prolonged, based on whether the activation is TRAF- or ROS-dependent (Nakano et al., 2006).

The correlation between ER stress and ROS generation has been well established in a number of studies and is related to cell homeostasis since OS is known to damage the structure of DNA and proteins directly due to oxidation and nitration, which induce ER stress (Saglar et al., 2014; Cai et al., 2018). In addition to protein damage and the increase in misfolded proteins, some studies have suggested that under normal conditions, the ER of eukaryotic cells has limited enzymatic antioxidant protection, which makes the ER especially vulnerable to oxidative stress (Santos et al., 2009; Cao and Kaufman, 2014). Studies have demonstrated that ER stress is induced by ROS (for example, in radiotherapy or chemotherapy), which potently upregulate UPR genes in lymphocytes, including XBP1, GRP78, ATF4, and CHOP (Hayashi et al., 2005). In fact, transgenic mice with overexpression of antioxidant enzymes (superoxide dismutase genes) had downregulated ATF4 and CHOP levels compared with those in wild-type animals (Li et al., 2015). Moreover, the increased stress level in cancer cells is the characteristic metabolic scenario that differentiates them from normal cells.

**FIGURE 7:**
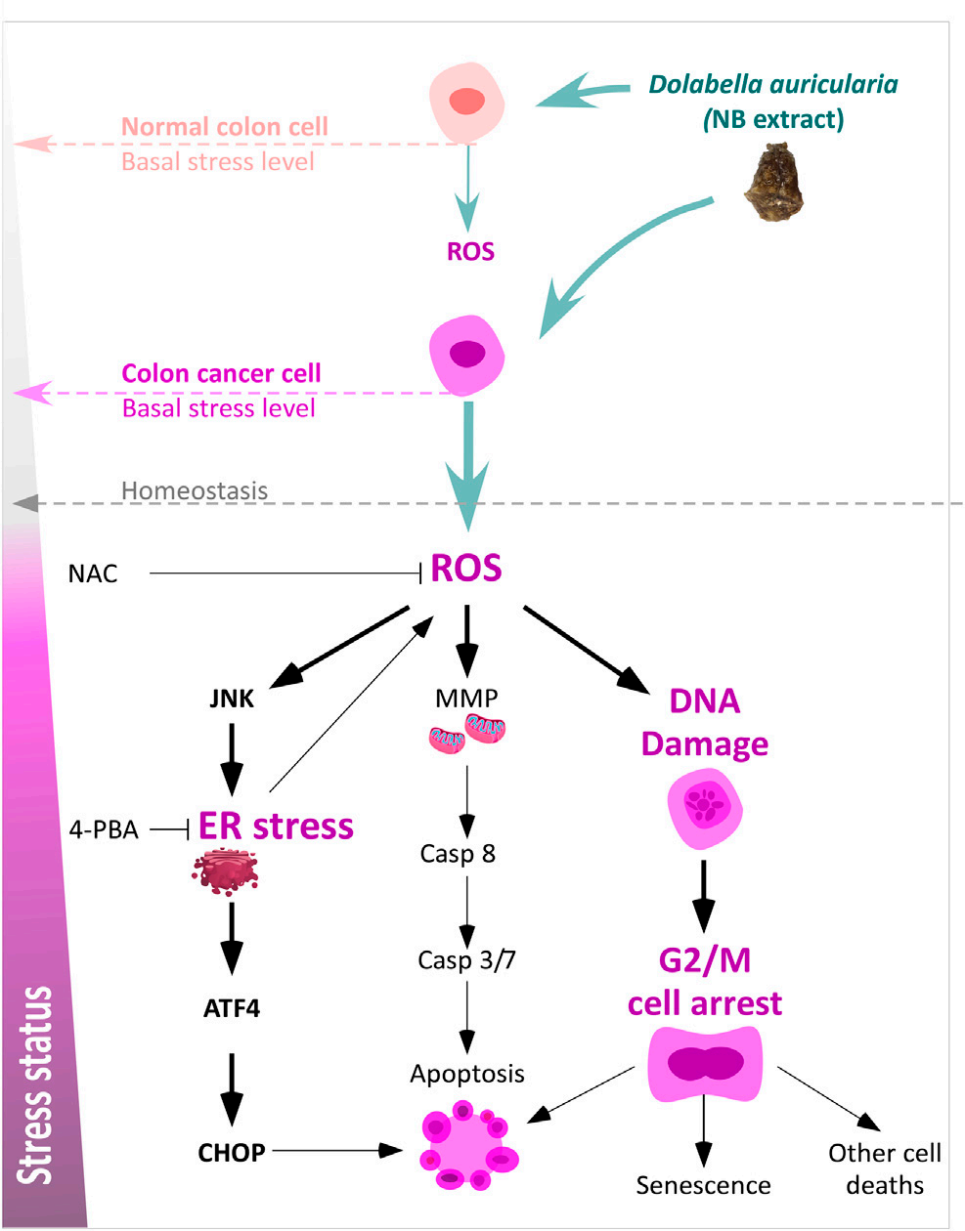
Schematic diagram of the effect of NB extract on colon cancer cells including an increase in OS and ER stress and the different effect in normal colon cells associated with the basal stress levels.

Our findings indicated that the ER stress response is activated by NB extract accompanied by mitochondrial dysfunction (Supplementary Figure S12) and DNA damage due to an increase in the intracellular ROS levels in CRC HCT-116 cells (Figure 7). Many studies have described a direct relationship between ER stress and cell death induction (Kim et al., 2008; Sano and Reed, 2013; Sovolyova et al., 2014; Li et al., 2015). Thus, drugs or compounds that are able to induce apoptotic cell death through ER stress may be considered an effective strategy to eliminate cancer cells (Sun et al., 2004). NB extract treatment was associated with activation of caspases and increased apoptosis; however, the arrest in the G2/M phase is the most remarkable effect after treatment of colon cancer cells with NB extract. The reason for the induction of significant cell arrest in the G2/M phase by NB extract, rather than a significant increase in cell death, can be due to the type and magnitude of induced ER stress and OS. Cell fate depends on the strength and nature of the OS and ER stress, which can determine whether cells can prolong the ER stress or undergo cell death (Wei et al., 2012; Cao and Kaufman, 2014). Thus, we suggest that NB extract leads to the production of ROS in colon cancer, which can disturb homeostasis by interfering with the protein folding process, inducing ER stress, and damaging lipids and DNA via oxidation or nitration (Karpinska and Gromadzka, 2013). This damage can be strong enough to induce checkpoint kinase 1 activation and G2/M arrest in colon cancer cells through the activation of the ataxia-telangiectasia mutated (ATM) protein kinase when DNA damage is not repaired. The G2 checkpoint prevents cells with damaged DNA from entering mitosis and leads to the suppression of proliferation (He et al., 2011).

The presence of OS and ER stress events as a consequence of NB extract treatment in the colon cancer cell line HCT-116 was demonstrated; in the present study, the order of events was established to determine whether OS or ER stress is upstream. If ROS are the trigger of the NB extract effect, then reductions in ER stress markers, DNA damage, G2/M arrest, and apoptosis are expected after NAC coincubation with NB extract. However, if ER stress is the main cause, then 4-PBA is expected to decrease the levels of these markers to a higher extent than that induced by NAC. ROS and ER stress are closely related; thus, it is possible that the NB extract effect induces ROS and ER stress simultaneously. In this case, the combination of NAC and 4-PBA with NB is expected to have the highest reduction effect. Our results demonstrate that NAC was able to reduce cell death and the expression of ER stress–related markers (phospho-IRE1α, phospho-JNK, ATF4, and CHOP) activated by NB extract in colon cancer HCT-116 cells. Therefore, we postulate that ROS are the main inductor of the imbalance in stress caused by NB extract. However, although DNA damage was slightly decreased by this antioxidant, G2/M cell cycle arrest was not inhibited. Thus, these results suggest that some compounds from NB extract can induce DNA damage and G2/M cell arrest in colon cancer cells regardless of the generated oxidative stress.

Our data clearly demonstrate that NB extract induces higher levels of intracellular ROS accumulation and ER stress in the colon cancer cells than those in the normal CCD-18Co colon cell line. The elevated stress levels induced by NB are quite different according to the comparison of the cancer and normal cells since CRC HCT-116 cells were more vulnerable to NB extract (Figure 7). This hypothesis was corroborated by an increase in the stress levels in the superinvasive HCT-116 populations. Many studies have reported higher levels of stress (OS and ER stress) in tumor cells than in normal cells (Pelicano et al., 2004; Wang et al., 2005; Dejeans et al., 2012). Cancer cells isolated from blood and metastatic sites were reported to have higher levels of ROS than those in primary subcutaneous tumors (Piskounova et al., 2015). An increase in the burden of OS can be explained by the development of an extra antioxidant capacity, which allows cells to continue proliferating (Gorrini et al., 2013) and is considered the source of new tumor-initiating cells [36]. Our data indicated that the ROS and ER stress levels were higher in the HCT-116 populations. Interestingly, NB extract was able to inhibit the proliferation, migration, and invasion CI of the most invasive HCT-116 populations at lower concentrations compared to its effect on the parental clones. Cancer cells can withstand higher levels of stress that is compensated for by an increase in the antioxidant system; however, this scenario makes them more sensitive to external stress-producing stimuli. According to Perillo B. et al., (Perillo et al., 2020), ROS can become Trojans horses that can be used to eliminate tumor cells. This feature has been recently used to target drugs and to develop a number of anticancer drugs.

Many types of cancer have been shown to be dependent on redox-regulating mechanisms, such as the glutaredoxin and thioredoxin systems, and are therefore vulnerable to changes and offer a promising anticancer target (Trachootham et al., 2009; Hegedus et al., 2018; Postovit et al., 2018; Mohammadi et al., 2019). Our results indicate that CRC HCT-116 cells have physiological adaptive mechanisms developed to survive in the overstressed state. However, when stress is stimulated over the threshold, which seems to correspond to the effect induced by NB extract, homeostasis is disturbed, triggering DNA damage and the mechanisms that will arrest the cell cycle to the point of no return, resulting in cell death. Various ROS-related effects that may compromise cancer cell viability include programmed cell death (apoptosis, necrosis, or autophagy), multidrug resistance inhibition, or epigenetic control by nuclear ROS (Perillo et al., 2020). NB extract can promote colon cancer cell death via oxidative stress–induced ER stress, followed by cell cycle arrest and apoptosis. Nevertheless, the exact mechanism of action of NB extract–induced generation of intracellular ROS in colon cancer cells should be further investigated to develop a potential ROS-related anticancer therapeutic strategy. These results indicate that compounds from *D. auricularia* extract, which have already been identified in a previous study (Ruiz-Torres et al., 2019), deserve additional investigation as natural chemosensitizing and anticancer agents.

## Conclusion

The extract derived from *Dollabella auricularia* exerted antiproliferative, antimigratory, and anti-invasive effects by inducing oxidative stress, overstressing the ER, and efficiently sensitizing human colon cancer cells with respect to less sensitive normal colon cells.

## Data Availability

The original contributions presented in the study are included in the article/Supplementary Material; further inquiries can be directed to the corresponding author.
