# Supplementary material for: A Nudibranch Marine Extract Selectively Chemosensitizes Colorectal Cancer Cells by Inducing ROS-Mediated Endoplasmic Reticulum Stress
Source: Front Pharmacol. 2021 Apr 8;12:625946. doi: 10.3389/fphar.2021.625946 (PMC8388012; doi:10.3389/fphar.2021.625946)
Supplement: Supplementary file 1 [file datasheet1.zip › Additional/VRT_Frontiers_Suplementary.docx]

**A nudibranch marine extract chemosensitizes selectively colorectal cancer cells by inducing**

**ROS-mediated endoplasmic reticulum stress**

Verónica Ruiz-Torres ^1^, Nicholas Forsythe ^2^, Almudena Pérez-Sánchez^1^, Sandra Van Schaeybröeck ^2^, Enrique Barrajón-Catalán^1,*,#^ and Vicente Micol ^1,3,#^.

1  Instituto de Investigación, Desarrollo e Innovación en Biotecnología Sanitaria de Elche (IDiBE), Universitas Miguel Hernández; 03202, Elche, Spain.

2 Drug Resistance Group, Centre for Cancer Research and Cell Biology, School of Medicine, Dentistry and Biomedical Science, Queen's University Belfast, Belfast, United Kingdom.

3 CIBER, Fisiopatología de la Obesidad y la Nutrición, CIBERobn, Instituto de Salud Carlos III., Palma de Mallorca 07122, Spain (CB12/03/30038).

* Correspondence: e.barrajon@umh.es; Tel.: +34-965-222-586

# Both authors share senior co-authorship.

**Supplementary Information**

1. **Densitometric analysis of western-blot from Figure 2 e and f (manuscript).**

A significant reduction of cell viability of HCT-116 and CCD-18Co, coupled with a high increase in intracellular ROS by the NB extract was demonstrated. Owing to the link between ROS and Endoplasmic Reticulum Stress (ER stress), some of the ER stress markers were studied under NB effect using a dose-manner in HCT-116 cell line served as a initial approach. In this section is shown the densiometric analysis from **Figure 2 e** in the manuscript (**Supplementary Figure 1**). NB induced a significant increae in ATF4 and CHOP showing statistical significances at the highest concentrations compared to untreated cells (control, C). NB increased the expression of ATF4 (6-fold at 10 µg/mL and 10-fold at 25 µg/mL) and CHOP (6-fold at 10 µg/mL and 8-fold at 25 µg/mL). Even though it was no expected, espression of sXBP1 was reduced by NB treatment. PARP cleaveage was used as an indicator of cell death in western blot analysis. NB increased PARP cleaveage (7-fold at 10 µg/mL and 8-fold at 25 µg/mL) with statistical significances indicanting the induction of apoptosis. On the basis of the above findings, 10 µg/mL of NB was considered the effective concentration selected for further assays.


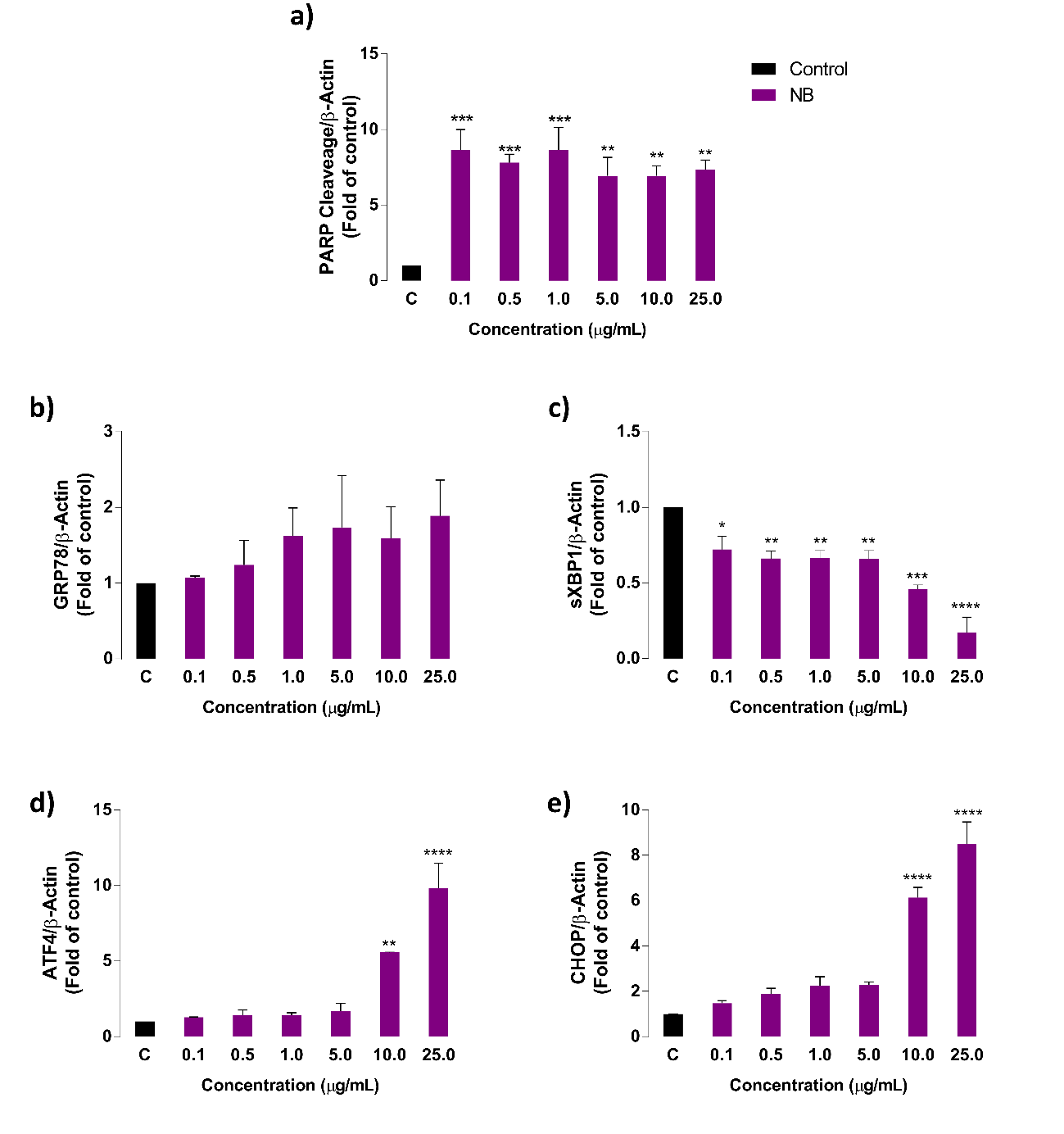


**Supplementary Figure 1. Relative protein expresion of ER stress markers in the colon cancer cell line HCT-116 under NB treatment in a dose-dependent manner obtained from densitometric measurement from western blot images in Figure 2 e.** ER stress proteins analyzed were GRP78 (**b**), sXBP1 (**c**), ATF4 (**d**), CHOP (**e**) and PARP (**a**) as an apoptosis indicator. Amounts of β-actin were used as internal loading controls. Data shown are mean ± SD at least of two independent experiments. Statistical significance are indicated as follows: * *p*-value<0.05, ** *p*-value<0.01, *** *p*-value<0.001 and **** *p*-value<0.0001 vs. control.

Next step was to study in depth the NB effect at 10 µg/mL in a time-dependent manner and to broaden the ER stress-related proteins tested in HCT-116 cell line (**Figure 2 f** in the manuscript and densiometric analysis in **Supplementary Figure 2)**. Results showed an early activation with statistical significances of phospho-JNK (4-fold), ATF4 (3-fold), phospho-eIF2α (1.6-fold) and CHOP (2-fold) after 3 h of NB treatment. At 6, 12 and 24 h of NB treatment ATF4 and CHOP were strongly upregulated. Phospho-IRE1α and phospho-eIF2α showed an interesting time-dependent activation from 3 to 6 h following NB treatmen, however significant differences were minimal. PARP was significant cleaveaged after 6 h of NB treatment and becomes more pronounced at 24 h after NB treatment.


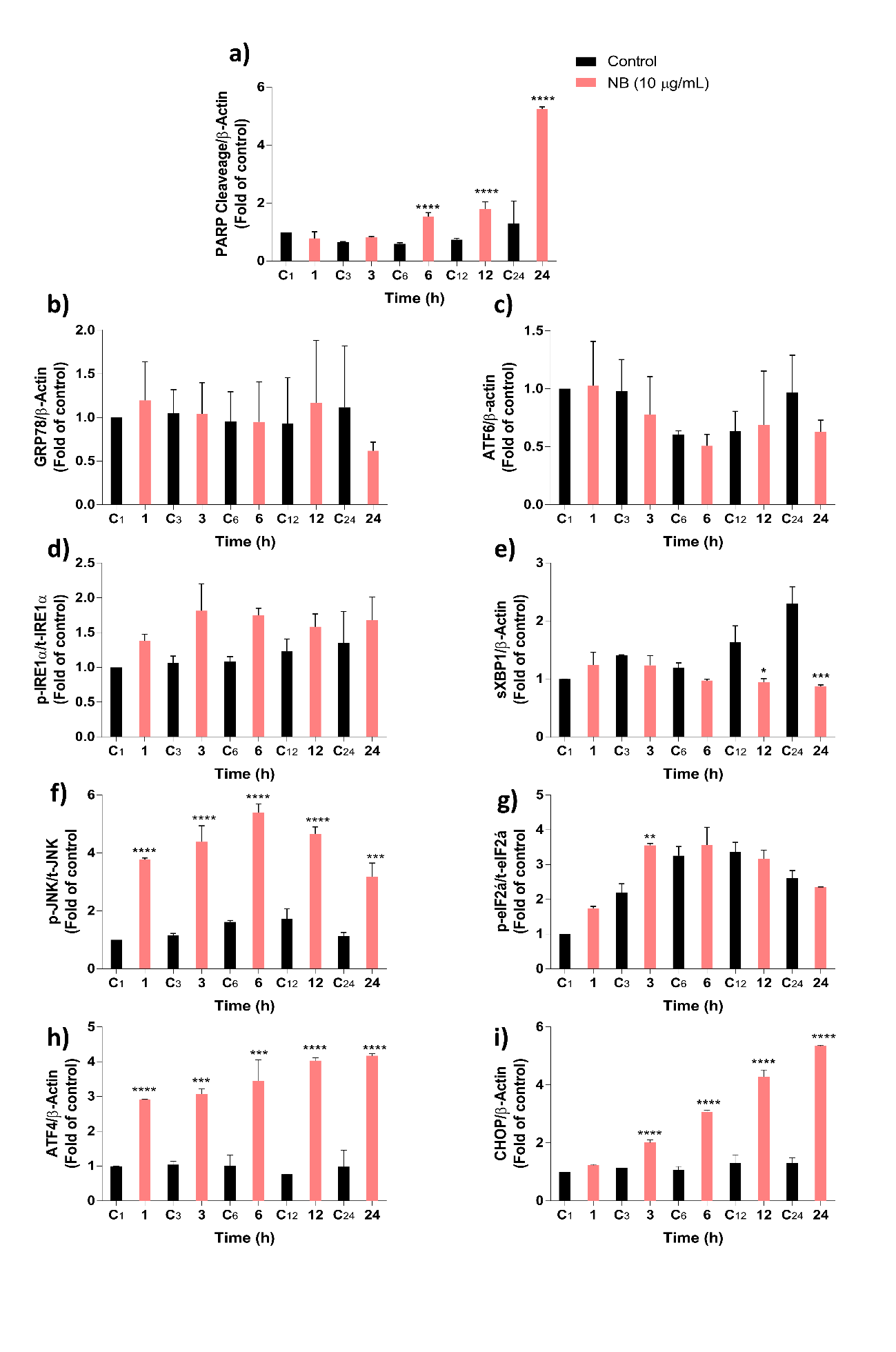


**Supplementary Figure 2. Relative protein expresion of ER stress markers in the colon cancer cell line HCT-116 under NB treatment at 10 µg/mL in a time-dependent manner obtained from densitometric measurement from western blot images in Figure 2 f.** ER stress proteins analyzed were GRP78 (**b**), ATF6 (**d**), phospho-IRE1α/total-IRE1α (**d**), sXBP1 (**e**), phospho-JNK/total-JNK (**f**), phospho-eIF2α/total-eIF2α (**g**), ATF4 (**h**) and CHOP (**i**). PARP (**a**) was analyzed as an apoptosis indicator. Amounts of β-actin were used as internal loading controls. Data shown are mean ± SD at least of two independent experiments. Statistical significance is indicated as follows: * *p*-value<0.05, ** *p*-value<0.01, *** *p*-value<0.001 and **** *p*-value<0.0001 vs. control.

1. **Densitometric analysis of western-blot from Figure 3 e and f (manuscript).**

To study the contribution of NB extract on the apoptotic cell death, we pre-incubated HCT-116 cells with the pan caspase z-VAD inhibitor and afterwards cells were treated with the NB extract at 10 µg/mL for 24 and 48 h. Expression of ER stress-markers was compared to the PARP cleavage by western blot (**Figure 3 e** in the manuscript and **Supplementary Figure 3**). Results showed PARP cleavage induced by NB extract at 24 and 48 h and it was recovered under z-VAD condition (a 0.8-fold at 24 h and 0.6-fold at 48 h) pointing caspases provide a part in the process of cell death. How ER stress markers are involved in cell death induced by NB? In spite of phospho-IRE1α, phospho-JNK and ATF4 are activated only CHOP was reduced under z-VAD condition at 48 h.

**
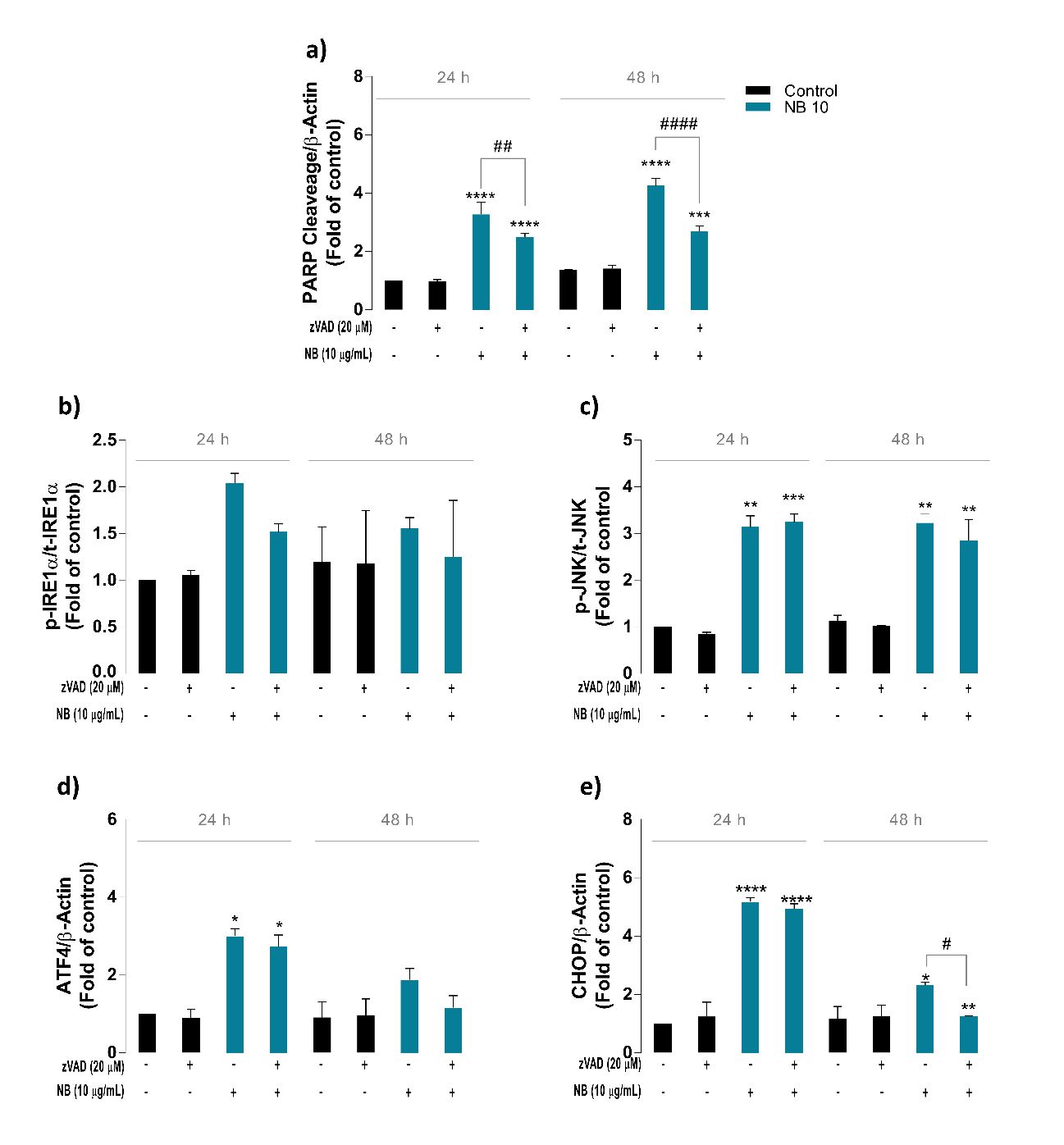
**

**Supplementary Figure 3. Analysis of the relation between ER stress and caspase-dependent apoptosis cell death in HCT-116 cells treated with *Dolabella auricularia* extract (NB) obtained from densitometric measurement from western blot images in Figure 3 e.** Cells were pretreated with the pan-caspase inhibitor, z-VAD (20 μM), for 2 h prior to the addition of NB extract at 10 μg/mL for 24 h. Expression of ER stress-related proteins were analyzed by western blot. Phospho-IRE1α/total-IRE1α (b), phospho-JNK/total-JNK (c), ATF4 (d) and CHOP (e). PARP (a) was analyzed as an apoptosis indicator. Amounts of β-actin were used as internal loading controls. Data shown are mean ± SD at least of two independent experiments. Statistical significance is indicated as follows: * *p*-value<0.05, ** *p*-value<0.01, *** *p*-value<0.001 and **** *p*-value<0.0001 vs. control and ^#^ *p*-value<0.05,^##^ *p*-value <0.01, ^###^ *p*-value<0.001 and ^####^ *p*-value<0.0001 vs. NB condition.

In addition, the type of apoptosis induced by NB at 24 h and 48 h was analysed silencing C8 (extrinsic apoptosis) and C9 (intrinsic apoptosis) using specific siRNAs (western blot **Figure 3 f** in the manuscript and densiometries in **Supplementary Figure 4**). NB induced PARP cleaveage at 24 h (5-fold) and 48 h (11-fold). At 24 h PARP cleaveage under NB effect was reduced with statistical significances when C8 was silenced (a 0.3-fold) indicating an early extrinsic apoptosis. However, at 48 h although PARP cleaveage induced by NB was reduced when C8 and C9 was silenced, there were not statistical significances between NB condition to C8 and C9 plus NB. These results would be signalling an early extrinsic apoptosis coupled to other types of cell death. Activation of ER stress markers, such as phospho-IRE1α, phospho-JNK, ATF4 and CHOP were not changed under C8 and C9 silencing corroborating a non-dependence of these proteins with caspase activation and apoptosis.

**
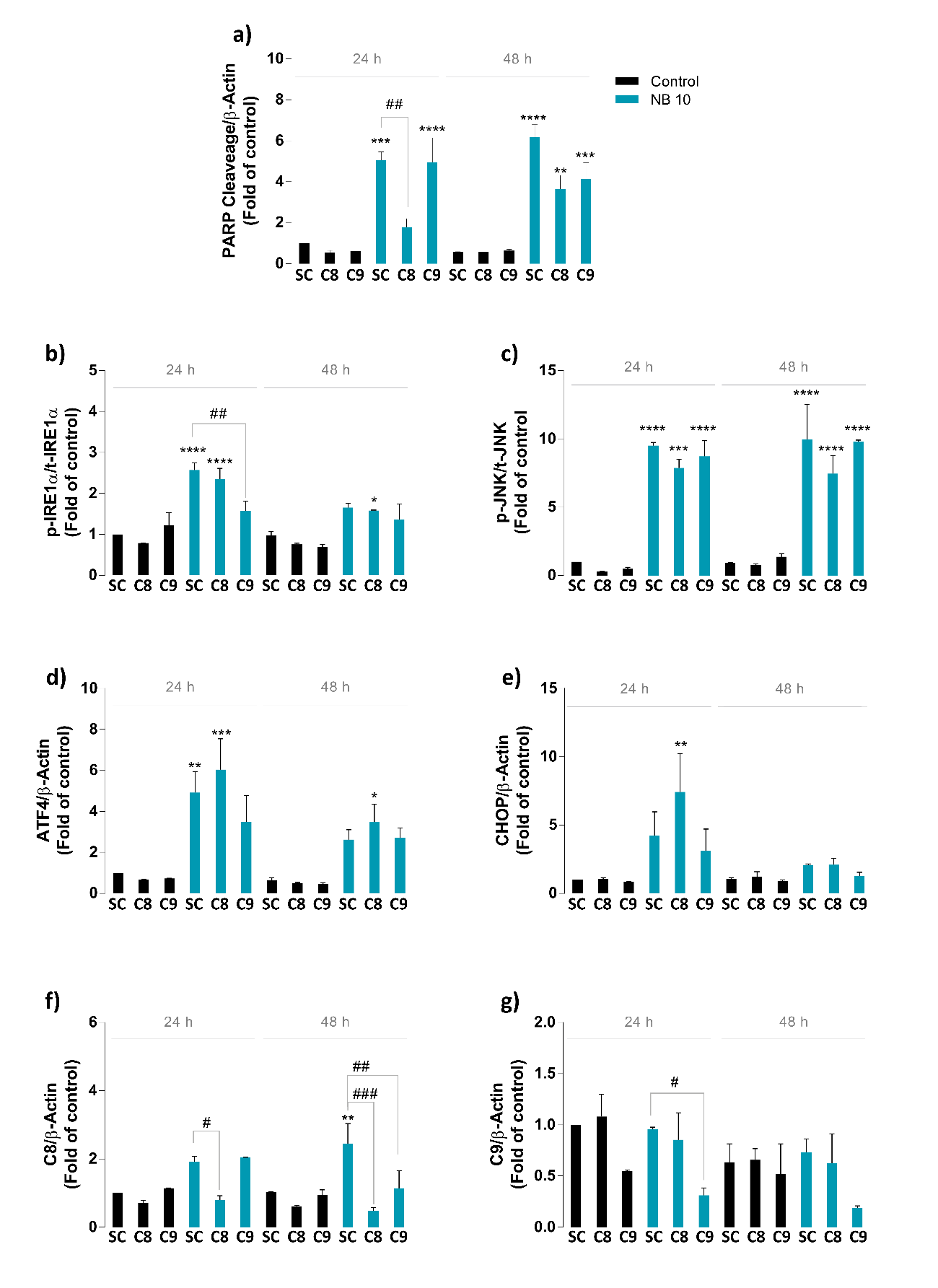
**

**Supplementary Figure 4. Analysis of the relation between ER stress and caspase8 and 9 dependent apoptosis cell death in HCT-116 cells treated with *Dolabella auricularia* extract (NB) obtained from densitometric measurement from western blot images in Figure 3 f.** Cells were pre-transfected with C8, C9 or non-targeting scrambled (SC) siRNA 24 h before the treatment with NB extract at 10 μg/mL for 24 and 48 h. Expression of ER stress-related proteins were analyzed by western blot. Phospho-IRE1α/total-IRE1α (b), phospho-JNK/total-JNK (c), ATF4 (d), CHOP (e), caspase 8 (f) and caspase 9 (g). PARP (a) was analyzed as an apoptosis indicator. Amounts of β-actin were used as internal loading controls. Data shown are mean ± SD at least of two independent experiments. Statistical significance is indicated as follows: * *p*-value<0.05, ** *p*-value<0.01, *** *p*-value<0.001 and **** *p*-value<0.0001 vs. control and ^#^ *p*-value<0.05,^##^ *p*-value <0.01, ^###^ *p*-value<0.001 and ^####^ *p*-value<0.0001 vs. NB condition.

1. **Non-dependent caspase cell death induced by NB extract.**

Results from section 2.3.1 in the manuscript, indicated an additional non-caspase dependent cell death induced by NB extract. Therefore necrosis, necroptosis and autophagy were studied in HCT-116 colon cancer cell model after 24 h of NB extract treatment.

Necrosis following 24h NB treatment was studied using the lactate dehydrogenase (LDH) assay. LDH is an intracellular enzyme that is released into the extracellular space when the membrane is broken in the necrotic death [1]. Colon cancer cells were seeded at 5 × 10^3^/well into 96-well plates. After 24 h, cells were treated with NB extract at the different concentration for 24 h, then the supernatant was collected and used according to the manufacturer’s instruction (Roche Diagnostic Systems, Montclair, NJ, USA). The concentration of LDH was measured with a microplate reader (SPECTROstar Omega, BMG Labtech, Germany) at a wavelength of 490 nm. Results are shown in Supplementary Figure 5 a.

The measurement of LDH in HCT-116 supernatants showed that NB did not increase LDH compared to negative and positive control after NB treatment (**Supplementary Figure 5 a**), therefore necrosis was discarded to take place among the putative cell death mechanisms.

Necroptosis is programmed cell death independent of caspases activity [2, 3] that can exposure PhS. For this reason, necroptosis was considered as a possible mechanism of NB-induced cell death. Cells were pretreated with the necroptotic inhibitors necrostatin-1 (nec-1, an RPK1 inhibitor [4]) at 40 µM and necrosulfonamide (nsa, an MLKL inhibitor [5]) at 0.5 µM for 1 h and z-VAD for 6 h and then treated with NB extract (10 μg/mL) for 24 h. Annexin V assay showed an increase in the apoptotic proportion to 14.0 ± 1.4 % following NB extract. Statistically significant differences were found only in the condition of z-VAD where early apoptotic population decreased to 8.5 ± 1.1 % (0.6-fold) (**Supplementary Figure 5 b**) suggesting that no necroptosis implication was present on the cell death mechanism. These results were supported after the G2/M phase analysis (**Supplementary Figure 5 c**), which not only showed that the effect of NB in G2/M arrest cell population was non-dependent on necroptosis but also that the main mechanism of G2/M arrest was not caspases-dependent as only a small, but significant decrease was obtained in the presence of z-VAD.

We have previously shown that a nudibranch extract from *Phyllidia varicose* inhibited phospho-mTOR (molecular target of rapamycin) related to autophagy [6]. mTOR appears to be deregulated in many cancers and its associated with cell proliferation and cancer progression [7]. According to these previous data, NB extract could also be inhibiting mTOR target and inducing autophagy. We investigated if the induction of autophagy would be a key event in mediating the effects observed following NB treatment, by using the autophagic inhibitor chloroquine (chlo). HCT-116 cells were pretreated with the autophagy inhibitor chloroquine (chlo) (10 μM) and the pan-caspase inhibitor, zVAD (20 μM) 2 h prior to the addition of NB extract at 10 µg/mL for 24 h. However, chlo did not affect the apoptotic population, as measured by PI staining, following NB treatment (**Supplementary figure 5 d**). In addition, the percentage of cells in the G2/M phase following NB treatment did not change with the addition of chlo (**Supplementary Figure 5 e**).


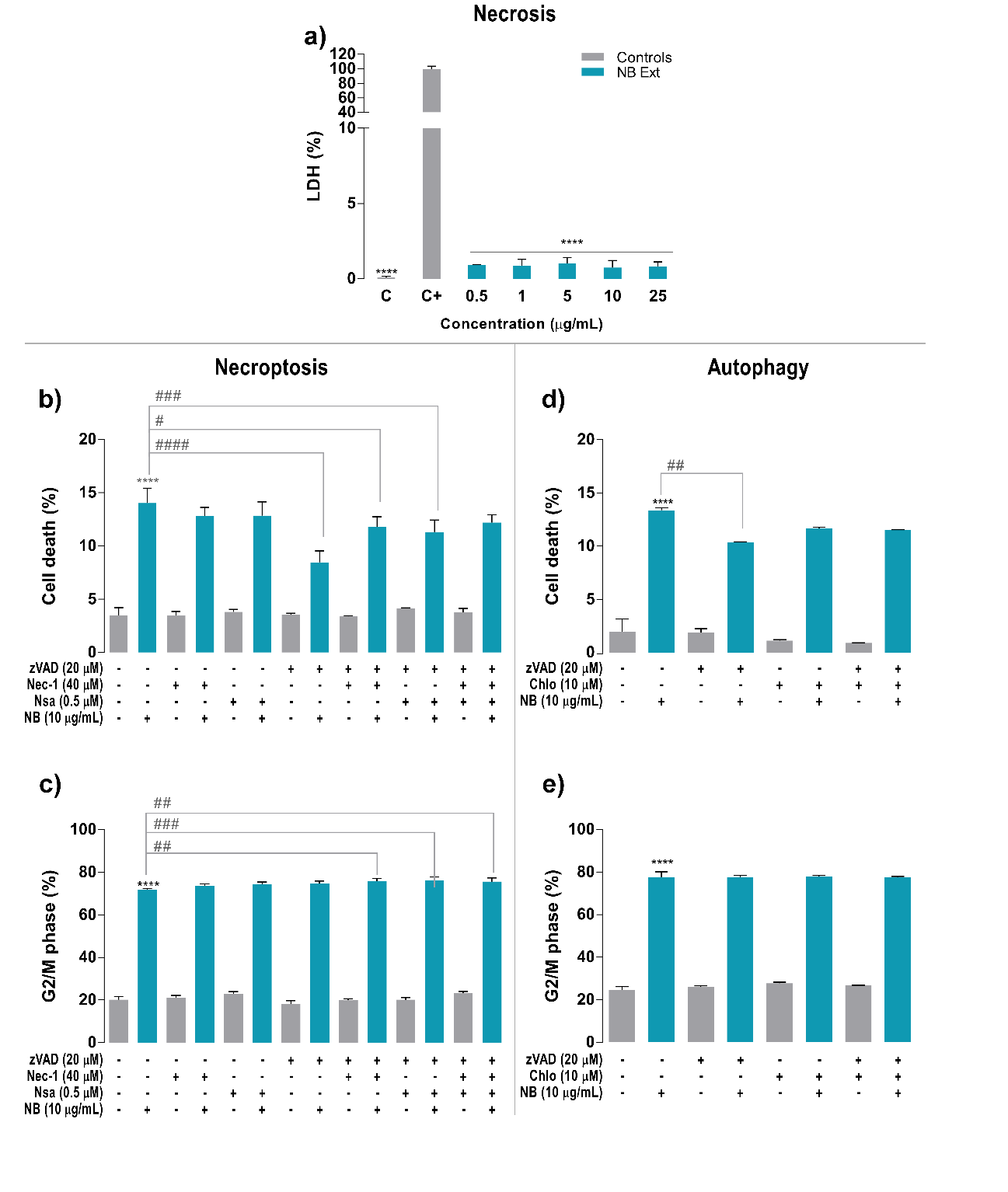


**Supplementary figure 5. Effect of NB extract on non-dependent caspase cell death (necrosis, necroptosis, and autophagy) in colon cancer HCT-116 cells.** HCT-116 cells were treated with NB extract at 0.5, 1, 5, 10 and 25 μg/mL for 24 h. Lactate dehydrogenase enzyme (LDH), was determined as necrosis indicator **(a)**. Data were compared to a positive control (lysis control, 100 % of LDH, C+) and to the negative control (untreated cells plus dimethyl sulfoxide less than 0.2 %, C-). HCT-116 cells were pretreated with the necroptosis inhibitors Necrostatin 1 (Nec-1) (40 μM) and Necrosulfonamide (nsa) (0.5 μM), and the pan-caspase inhibitor, z-VAD (20 μM), 2 h prior to the addition of NB extract at 10 μg/mL for 24 h. Cell death **(b)** and G2/M phase **(c)** was measured using Propidium iodide (PI) through flow cytometry. HCT-116 cells were pretreated with the autophagy inhibitor chloroquine (chlo) (10 μM) 2 h prior to the addition of NB extract at 10 μg/mL for 24 h. Cell death **(d)** and G2/M **(e)** was measured using Propidium iodide (PI) through flow cytometry. Data were represented as mean ± SD from three independent experiments. *p*-values were calculated and compared to the untreated cell line (*****) and NB condition (**#**) using ANOVAs. * ^or #^ *p*-value< 0.05, ** ^or ##^ *p*-value< 0.01, *** ^or ###^ *p*-value< 0.001 and ****^or ####^ *p*-value< 0.0001.

1. **Densitometric analysis of western-blot from Figure 4 a (manuscript).**

To study the relation between oxidative stress (OS) and ER stress, the ROS scavenger N-acetyl cysteine (NAC, 5 mM) and the ER stress inhibitor 4-phenylbutyric acid (4-PBA, 5 mM) and its combination were used to pretreat for 2 h cells before adding NB extract at 10 μg/mL for 24 h. Effect of NB in the tumoral HCT-116 cell line was compared to the normal CCD-18Co. Western blots of HCT-116 are shown in **Figure 4 a**, densiometry analisis in **Supplementary Figure 6**. The PARP escision, induced by NB after 24 h (increased to 4-fold), is only recovered by the antioxidant NAC (reduced by 0.4-fold). On the contrary the ER stress inhibitor 4-PBA and its combination with NAC keep the PARP cleaveage or even increased it. Results obtained indicate a strong relation between the NB effect with an oxidant activity. Regarding to the ER stress markers, these results show a significant inhibition of ATF4 (0.6-fold) and CHOP (0.6-fold) when the scavenger NAC avoid ROS induced by NB. Phospho-IRE1α was considerably also reduced in the 4-PBA (0.6-fold) and 4-PBA plus NAC (0.4-fold) conditions. The remaining ER stress markers measured were not reduced when NAC,4-PBA or 4-PBA plus NAC were used. Thus, this results would be pointing the NB effect in CHOP and ATF4 related-ER stress proteins are highly connected to an imbalance in intracellular ROS.


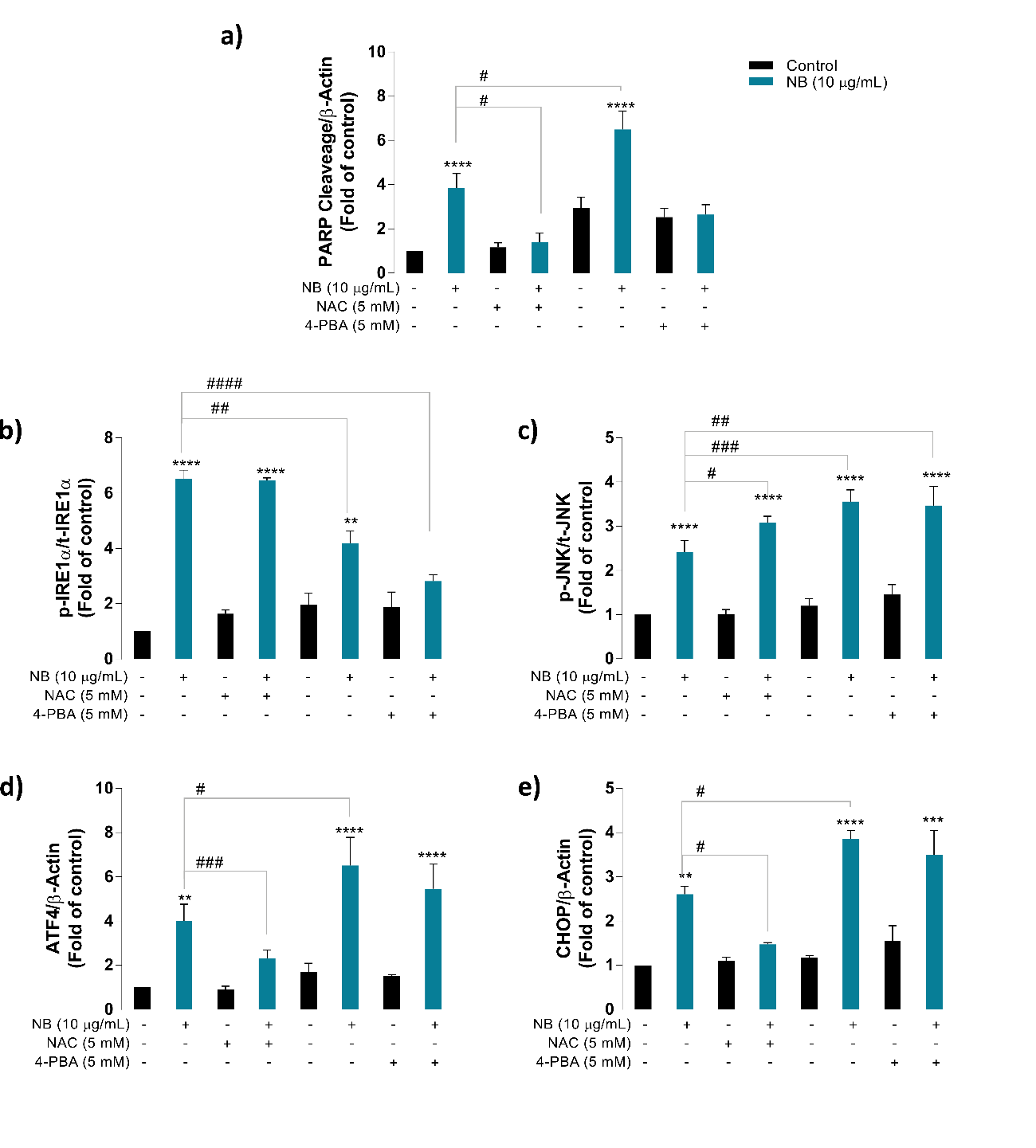


**Supplementary Figure 6. Analysis of the contribution of ER stress and ROS to the effect of NB extract in HCT-116 by densitometric measurement from western blot images in Figure 4 a.** Cells were pretreated with N-acetyl cistein (NAC, 5 mM), 4-phenyl butyric acid (4-PBA, 5 mM), for 2 h prior to the addition of NB extract at 10 μg/mL for 24 h. Expression of ER stress-related proteins were analyzed by western blot. Phospho-IRE1α/total-IRE1α (b), phospho-JNK/total-JNK (c), ATF4 (d) and CHOP (e). PARP (a) was analyzed as an apoptosis indicator. Amounts of β-actin were used as internal loading controls. Data shown are mean ± SD at least of two independent experiments. Statistical significance is indicated as follows: * *p*-value<0.05, ** *p*-value<0.01, *** *p*-value<0.001 and **** *p*-value<0.0001 vs. control and ^#^ *p*-value<0.05,^##^ *p*-value <0.01, ^###^ *p*-value<0.001 and ^####^ *p*-value<0.0001 vs. NB condition.

1. **Contribution of ROS and ER stress in the NB extract effect in the normal colon cell model CCD-18Co.**

The objective of this section is to verify whether both the increase in ROS and ER stress-related proteins produced by NB in ​​CRC take the same role in the normal colon cell model CCD-18Co. The ROS scavenger N-acetyl cysteine (NAC, 5 mM) and the ER stress inhibitor 4-phenylbutyric acid (4-PBA, 5 mM, [8]) were used to pretreat cells for 2 h before the exposure to the NB extract (10 μg/mL).

**Supplementary Figure 7 a (**densiometric analysis in **Supplementary Figure 8)** shows the non-activation of the ER stress-related proteins, as well as the cell death (PARP cleavage) after NB treatment of CCD-18Co cells.

By contrast to the effect of NB in the colon cancer cell model HCT-116, NB was not able to induce a significant apoptotic effect in normal cell line CCD-18Co (**Supplementary Figure 7 b**). Related to cell cycle, NB treatment arrested CCD-18Co in G/2M a 26.6 % vs 20.1 % in control, and NAC, 4-PBA and its combination were not able to reduce significantly (**Supplementary Figure 7 c**). ROS level induced by NB extract was less increased than in HCT-116 cells by NB at 10 µg/mL, and it was reduced by NAC, 4-PBA and its combination (**Supplementary Figure 7 d**). DNA damage measured by the activation of H2A.γ was also studied in the CCD-18Co model after NB treatment. NB induced the activation of H2A. γ (CONT: 1.2 ±0.7 – NB: 23.2 ± 0.8 %) and interestingly was reduced drastically under NAC condition, and slightly by 4-PBA and by the combination of both inhibitors (NB: 23.2 ± 0.8 % - NB+NAC: 1.9 ± 0.8 % - NB+4-PBA: 19.6 ± 1.4 % – NB+NAC+4PBA: 17.0 ± 0.8 %) (**Supplementary Figure 7 e**).


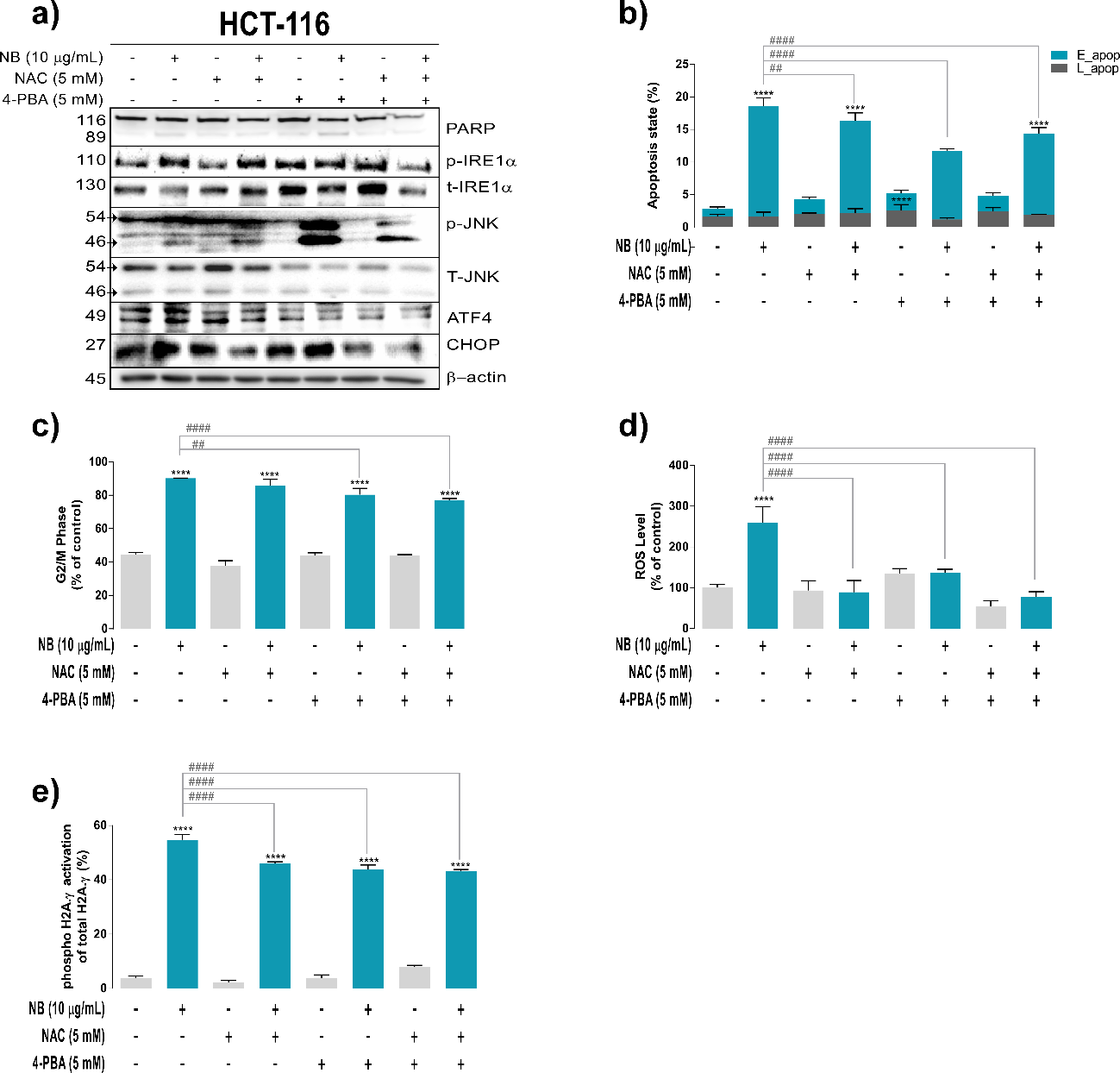


**Supplementary Figure 7. The relation between ROS and ER stress induced by NB extract in the normal colon cell model** **CCD-18Co.** Cells were pretreated with ROS scavenger N-acetyl-cysteine (NAC, 5 mM) and ER stress inhibitor 4-Phenylbutric acid (4-PBA, 5 mM) for 2 h prior to the NB treatment at 10 μg/mL. Ability of NB extract to modulate ER stress-related proteins (PARP, phospho-IRE1α, total-IRE1α, phospho-JNK, total-JNK, ATF4 and CHOP) was analyzed by western blot **(a)**. β-actin was used as a loading control. The experiment was repeated twice. Apoptosis **(b)**, G2/M phase **(c),** and phosphorylation of H2Aϒ (as DNA damage indicator) **(d)** were measured using the Muse® Cell Analyzer according to the manufacturer’s instructions. ROS levels were measured using dichloro-dihydro-fluorescein diacetate (H_2_DCF-DA) fluorescence label **(e)**. Data were represented as mean ± SD from three independent experiments. *p*-values were calculated and compared to the control (untreated cells plus dimethyl sulfoxide less than 0.2 %, C) (*****) and NB condition (**#**) using ANOVAs. * ^or #^ *p*-value< 0.05, ** ^or ##^ *p*-value< 0.01, *** ^or ###^ *p*-value< 0.001 and **** ^or ####^ *p*-value< 0.0001.

**
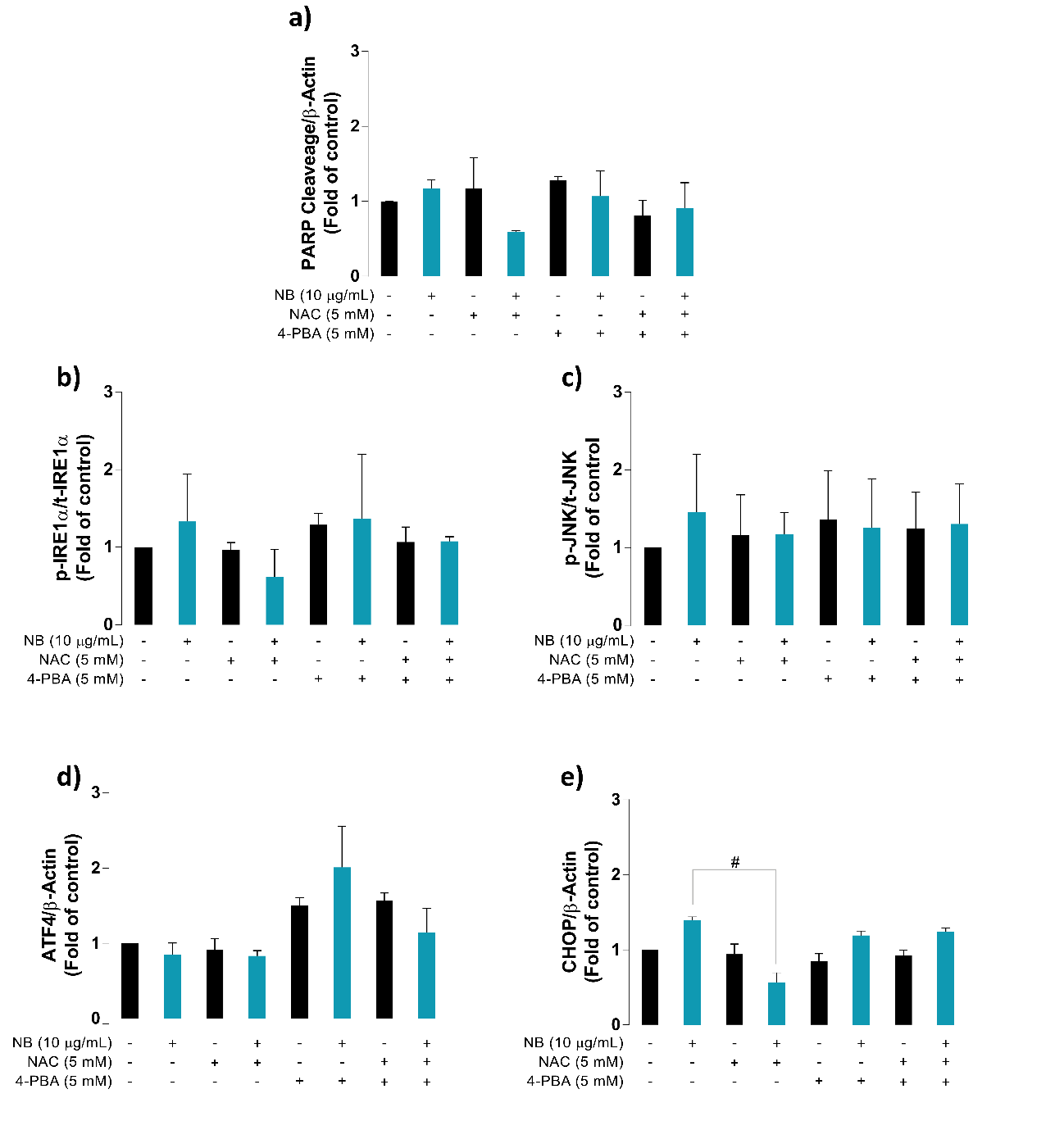
**

**Supplementary Figure 8. Analysis of the contribution of ER stress and ROS to the effect of NB extract in CCD-18Co by densitometric measurement from western blot images in Supplementary Figure 7.** Cells were pretreated with N-acetyl cistein (NAC, 5 mM), 4-phenyl butyric acid (4-PBA, 5 mM), for 2 h prior to the addition of NB extract at 10 μg/mL for 24 h. Expression of ER stress-related proteins were analyzed by western blot. Phospho-IRE1α/total-IRE1α (b), phospho-JNK/total-JNK (c), ATF4 (d) and CHOP (e). PARP (a) was analyzed as an apoptosis indicator. Amounts of β-actin were used as internal loading controls. Data shown are mean ± SD at least of two independent experiments. Statistical significance is indicated as follows: * *p*-value<0.05, ** *p*-value<0.01, *** *p*-value<0.001 and *p*-value<0.0001 vs. control and ^#^ *p*-value<0.05, ^##^ *p*-value<0.01, ^###^ *p*-value<0.001 and ^####^ *p*-value<0.0001 vs. NB condition.

1. **Densitometric analysis of western-blot from Figure 5 f (manuscript)**

In order to characterize superinvasive populations isolated from HCT-116 cell line, the basal expression of ER stress markers were measured by western blot in **Figure 5 f** and densiometries are in **Supplementary Figure 9**. Some differences were found between superinvasive I9 (I9), superinvasive I4 (I4)and parentals (P) HCT-116. A higher basal activation of phospho-JNK, ATF4 and CHOP were mesured in the most invasive phenotype of HCT-116. Phospho-JNK reached an increase of 1.5-fold in I4 and 1.7-fold in I9 respect to P. ATF4 showed a 1.1-fold in I4 and 1.9-fold in I9 higher than P and CHOP was found 1.8-fold in I4 and 2.7-fold in I9 higher respect to P. On the other hand, phospho-IRE1α was slightly reduced to a 0.8-fold in I4 and 0.7 in I9 in comparison to P. In the remaining ER stress proteins, although tendencies were found, there were not statistical differences respect to the P cell line. These results would be showing an over-activation of phospho-JNK, ATF4 and CHOP in superinvasive HCT-116 populations.

**
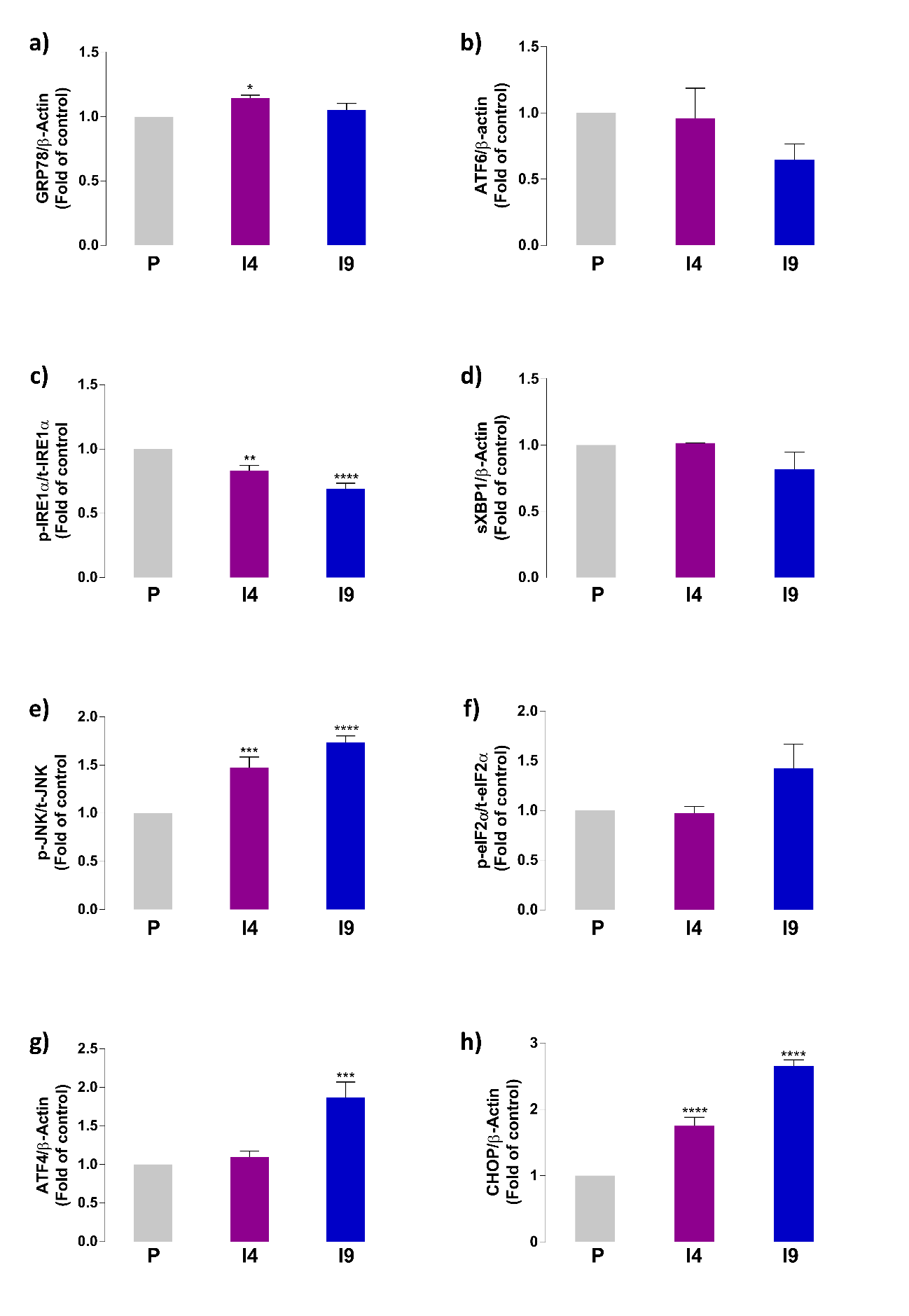
**

**Supplementary Figure 9. Characterization of ER stress related proteins of superinvasive populations of HCT-116 cell lines by densitometric measurement from western blot images in Figure 5 f.** HCT-116 parentals, HC-T116 superinvasive 4 (I4) and superinvasive 9 (I9) were compared. GRP78 (a), ATF6 (b), phospho-IRE1α/total-IRE1α (c), sXBP1 (d), phospho-JNK/total-JNK (e), phospho-eIF2α/ total-eIF2α (f), ATF4 (g) and CHOP (h). Amounts of β-actin were used as internal loading controls. Data shown are mean ± SD at least of two independent experiments. Statistical significance is indicated as follows: * *p*-value<0.05, ** *p*-value<0.01, *** *p*-value<0.001 and *p*-value<0.0001 vs. control.

1. **Densitometric analysis of western-blot from Figure 6 e and f (manuscript)**

The superinvasive HCT-116 population showed different levels os OS and ER stress. Next step was to test if NB extract induce the same effect in these populations. HCT-116 P, I4 and I9 were treated with NB at 10 µg/mL for 24 h and ER stress makers were analyzed by western blot (images are shown in **Figure 6 e** and densiometries in **Supplementary Figure 10**). NB induce higher cell death (PARP cleaveage) in the more invasive population of HCT-116. It was registered an increase of 2.5-fold in P, 2.4-fold in I4 and 1.9-fold in I9 treated with NB respect to untreatead populations. Furthermore, this effect was correlated to a more expression of phospho-JNK the more invasive population were. Data registered show an increase in phospho-JNK in 3.9-fold in P, 5.5-fold in I4 and 6.9-fold in I9 respect to untreatead populations. ATF4 and CHOP were hihghly activated by NB extract, however there was not a correlation between the activation and the invasive phenotype or it was not found a sensitive behavior dependent of the invasive phenotype. The increased ATF4 was 9.2-fold in P, 6.0-fold in I4 and 2.4-fold in I9 and CHOP was activated to 56.7-fold in P, 31.5-fold in I4 and 3.4-fold in I9 respect to untreatead populations.


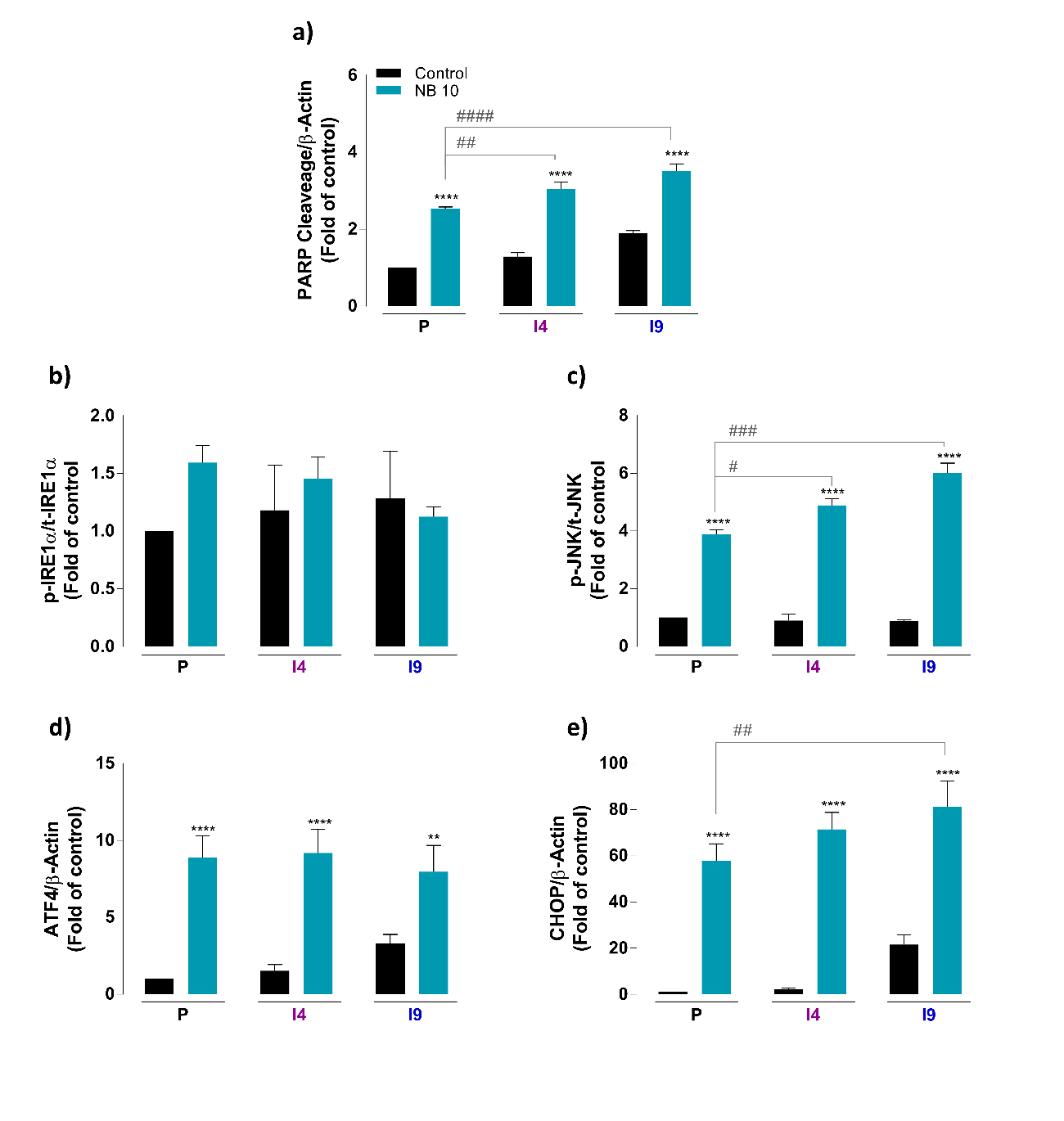


**Supplementary Figure 10. Relative protein expresion of ER stress markers of HCT116 P, I4 and I9 invasive populations under NB treatment at 10 µg/mL obtained from densitometric measurement of western blot images in Figure 6 e.** ER stress proteins analyzed were phospho-IRE1α/total-IRE1α (b), phospho-JNK/total-JNK (c), ATF4 (d) and CHOP (e). PARP (a) was analyzed as an apoptosis indicator. Amounts of β-actin were used as internal loading controls. Data shown are mean ± SD at least of two independent experiments. Statistical significance is indicated as follows: **p*-value<0.05, ** *p*-value<0.01, *** *p*-value <0.001 and *p*-value<0.0001 vs. control and ^#^ *p*-value <0.05, ^##^ *p*-value<0.01, ^###^ *p*-value<0.001 and ^####^ *p*-value<0.0001 vs.parental NB condition.

For the purpose of trying to understand the relation to ER stress and OS induced by NB and its anti-invasive effect, the superinvasive population HCT-116 I9 was pretreated with NAC and 4-PBA at 5 mM for 2 h prior to NB exposure for 24 h, and ER stress makers were analyzed by western blot (images in **Figures 6 f** and densiometries in **Supplementary Figure 11**). The PARP excision induced by NB (increased 5-fold) was only reduced under NAC (0.8-fold). The study of ER stress markers can elucidate which they trigger these events. Densiometries analyzes revealed only NAC was able to reduce phospho-JNK (0.6-fold), ATF4 (0.4-fold) and CHOP (0.3-fold) with statistical significances. Therefore, these findings give us cause to believe that the imbalance of ROS induced by NB extract iniciates ER stress leading a serie of process which end in an inhibition of invasiviveness.

**
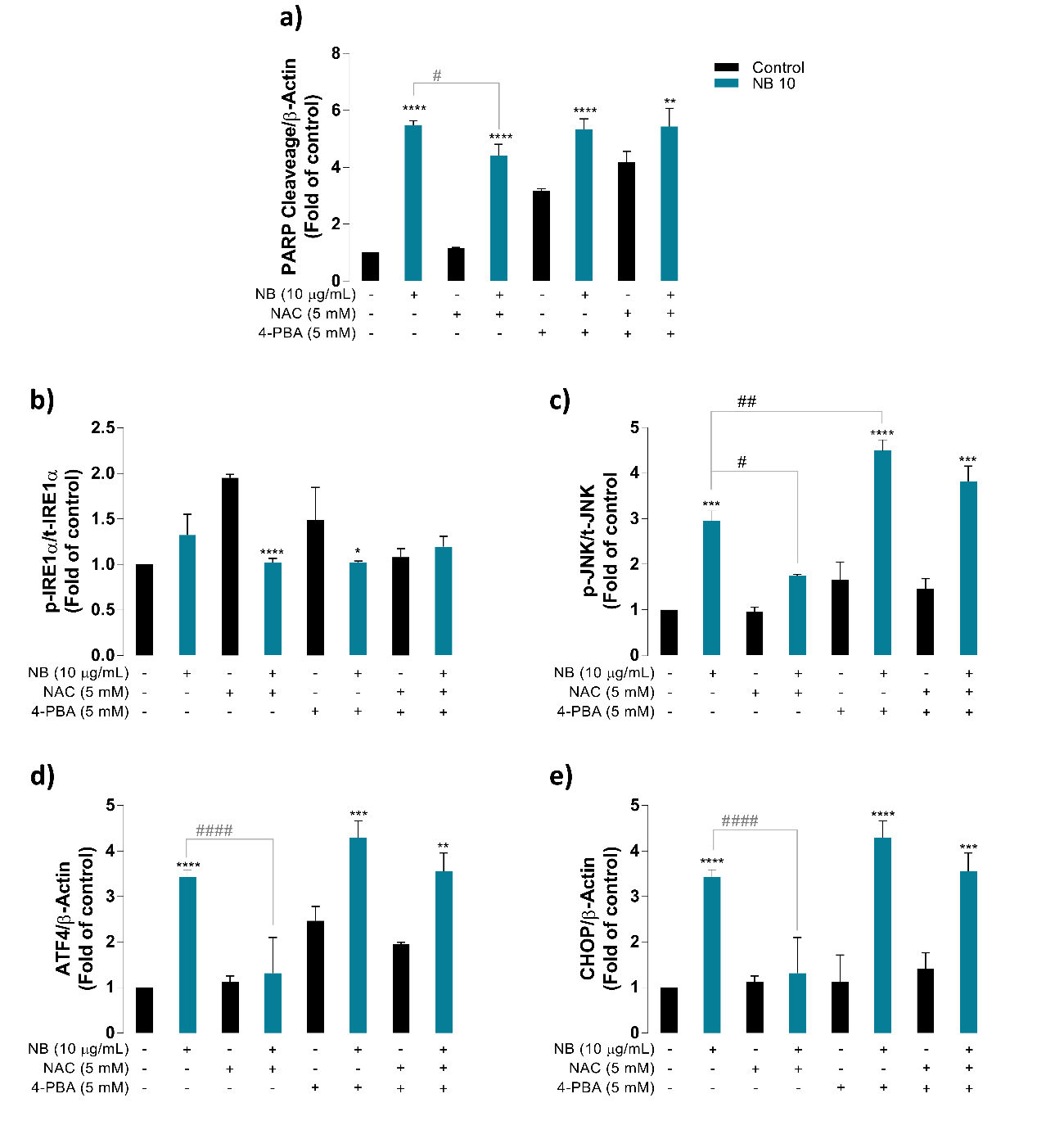
**

**Supplementary Figure 11. Analysis of the contribution of ER stress and ROS to the anti-invasive effect of NB extract in superinvasive population HCT-116 I9 cells by densitometric measurement from western blot images in Figure 6 f.** HCT-116 I9 subline was pretreated with N-acetyl cistein (NAC, 5 mM), 4-phenyl butyric acid (4-PBA, 5 mM) for 2 h prior to the addition of NB extract at 10 μg/mL for 24 h. Expression of ER stress-related proteins were analyzed by western blot. Phospho-IRE1α/total-IRE1α (b), phospho-JNK/total-JNK (c), ATF4 (d) and CHOP (e). PARP (a) was analyzed as an apoptosis indicator. Amounts of β-actin were used as internal loading controls. Data shown are mean ± SD at least of two independent experiments. Statistical significance is indicated as follows: **p*-value<0.05, ** *p*-value<0.01, *** *p*-value <0.001 and *p*-value<0.0001 vs. control and ^#^ *p*-value <0.05, ^##^ *p*-value<0.01, ^###^ *p*-value<0.001 and ^####^ *p*-value<0.0001 vs.parental NB condition.

1. **Mitochondrial Membrane Potential (MMP) analysis using MitoTracker® dyes.**

Mitochondria are critical to cell survival. As an important feature of mitochondria function is the mitochondrial membrane potential (MMP) which depends on mitochondrial membrane permeability and the oxidation-reduction activity. When mitochondria are dysfunctional these play key role in activating apoptosis in mammalian cells [9]. Considering previous and H_2_DCF-DA results the effect of NB in MMP of HCT-116 as a mitochondrial membrane permeabilization and mitochondrial damage was developed. Cells were treated with NB extract at different concentrations (0.1, 0.5, 1, 5, 10 and 25 µg/mL) for 24 h and MMP was studied using the ratio between MitoTracker®Red (MitoRed) and the total mitochondrial content using MitoTracker®Green (MitoGreen) both fluorescence probes. NB extract decreased MMP of HCT-116 cells in a dose-dependent way, reducing in a 0.8-fold at the lowest concentration of 0.1 µg/mL (CONT: 100.0 ± 9.3 % - NB_0.1_: 83.1 ± 7.5 %), reaching the 0.4-fold of reduction at 25 µg/mL (CONT: 100.0 ± 9.3 % - NB_0.1_: 42.5 ± 2.8 %). These results indicate NB extract disrupt the mitochondrial membrane.

We found all doses of marine extracts increased ROS levels and loss of MMP. Our results indicate that marine extracts could be disrupting the mitochondrial homeostasis, increasing ROS accumulation and perturbing MMP in a dose-dependent manner.


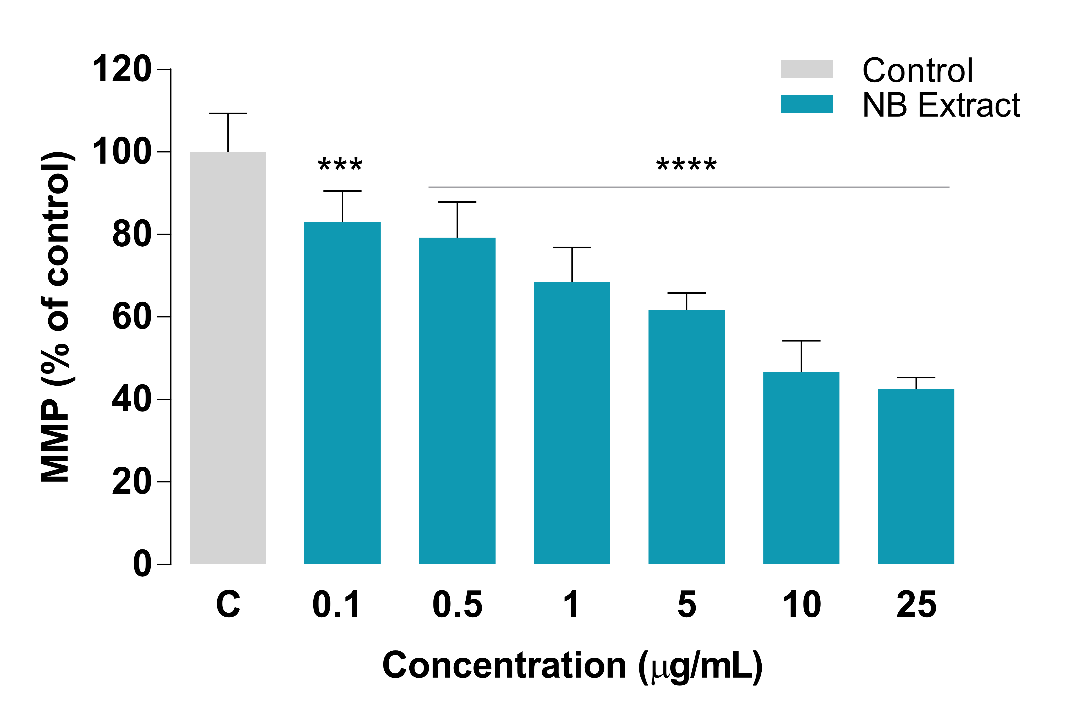


**Supplementary Figure 1****2. Effect of *Dolabella auricularia* extract (NB) on the the mitochondrial membrane polarization (MMP) in HCT-116 colon cancer cells.** Depolarization of the mitochondrial Membrane was measured normalizaing the MitoTracker Red CM-ROS signal respect to the MitoTracker® green fluorescence label in HCT-116 cells after 24 h of NB treatment at differente concentrations. Results were compared to untreated cells (Control cells plus dimethyl sulfoxide less than 0.2 %, C) and presented as the percentages (mean ± SD) from three independent experiments. *p-values* were calculated and compared to the same untreated cell line. **p*-value<0.05, ** *p*-value<0.01, *** *p*-value <0.001 and *p*-value<0.0001.

**Supplementary references**

1. Chan, F.K.-M., K. Moriwaki, and M.J. De Rosa, *Detection of necrosis by release of lactate dehydrogenase activity.* Methods in molecular biology (Clifton, N.J.), 2013. **979**: p. 65-70.

2. Sawai, H., *Characterization of TNF-induced caspase-independent necroptosis.* Leuk Res, 2014. **38**(6): p. 706-13.

3. Zargarian, S., et al., *Phosphatidylserine externalization, "necroptotic bodies" release, and phagocytosis during necroptosis.* PLoS Biol, 2017. **15**(6): p. e2002711.

4. Vandenabeele, P., et al., *Necrostatin-1 blocks both RIPK1 and IDO: consequences for the study of cell death in experimental disease models.* Cell death and differentiation, 2013. **20**(2): p. 185-187.

5. Sun, L., et al., *Mixed lineage kinase domain-like protein mediates necrosis signaling downstream of RIP3 kinase.* Cell, 2012. **148**(1-2): p. 213-27.

6. Ruiz-Torres, V., et al., *New Mammalian Target of Rapamycin (mTOR) Modulators Derived from Natural Product Databases and Marine Extracts by Using Molecular Docking Techniques.* Mar Drugs, 2018. **16**(10).

7. Populo, H., J.M. Lopes, and P. Soares, *The mTOR signalling pathway in human cancer.* Int J Mol Sci, 2012. **13**(2): p. 1886-918.

8. Sarvani, C., D. Sireesh, and K.M. Ramkumar, *Unraveling the role of ER stress inhibitors in the context of metabolic diseases.* Pharmacol Res, 2017. **119**: p. 412-421.

9. Handy, D.E. and J. Loscalzo, *Redox regulation of mitochondrial function.* Antioxid Redox Signal, 2012. **16**(11): p. 1323-67.
