# Supplementary figures and images for: A Nudibranch Marine Extract Selectively Chemosensitizes Colorectal Cancer Cells by Inducing ROS-Mediated Endoplasmic Reticulum Stress
Source: Front Pharmacol. 2021 Apr 8;12:625946. doi: 10.3389/fphar.2021.625946 (PMC8388012; doi:10.3389/fphar.2021.625946)

FIGURE 2 e

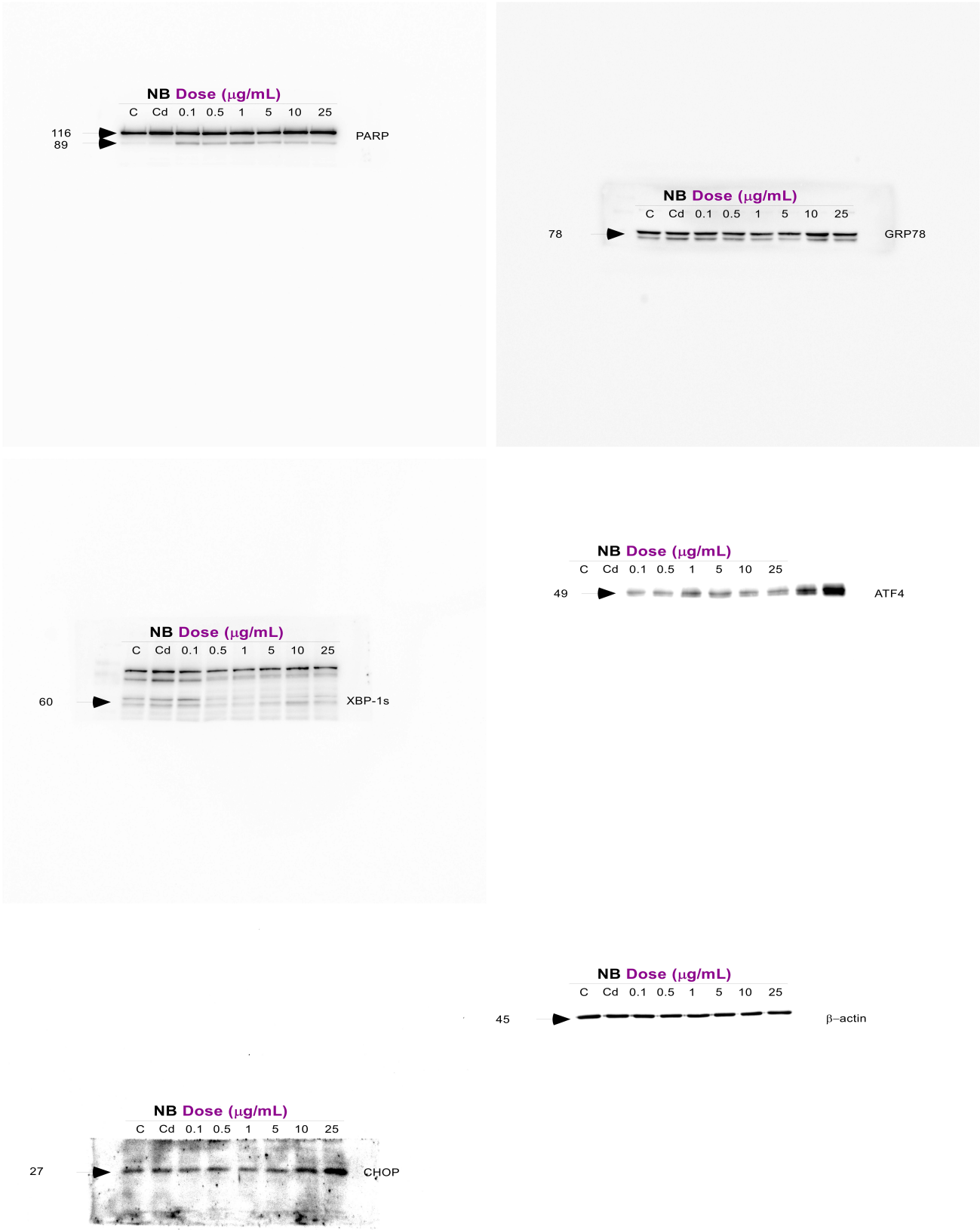

FIGURE 2 f

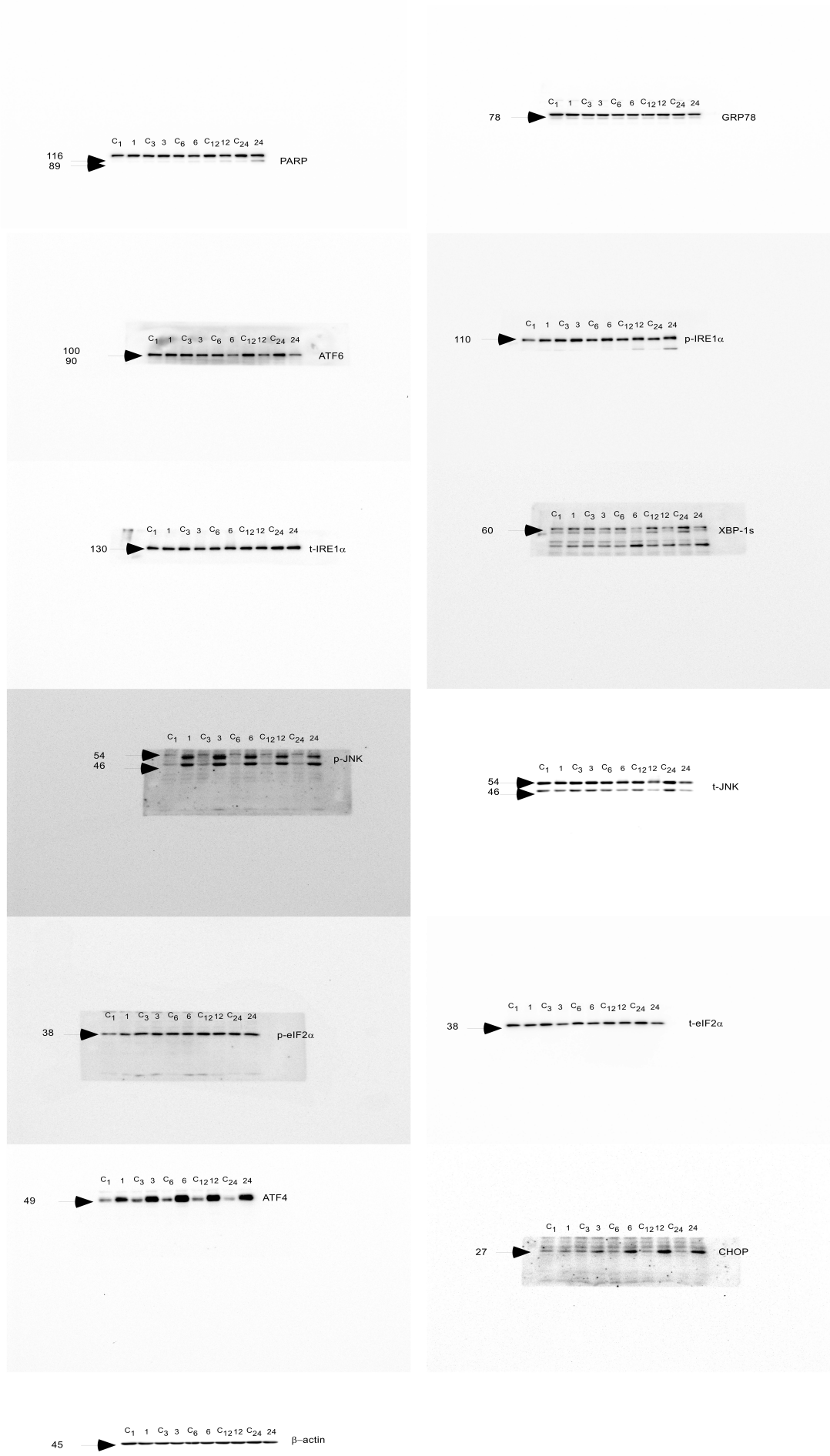

FIGURE 3 e

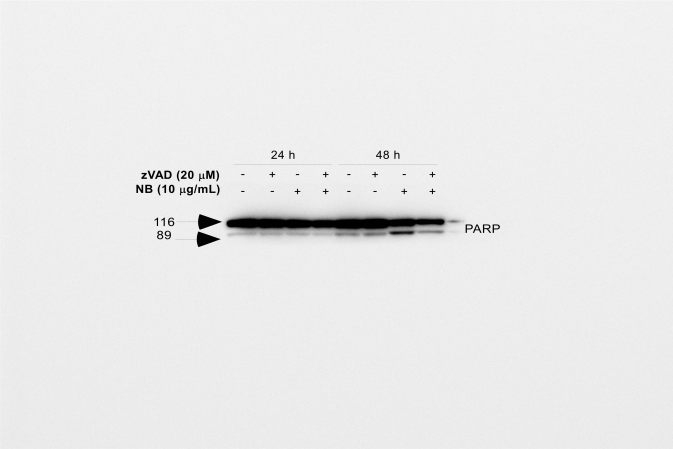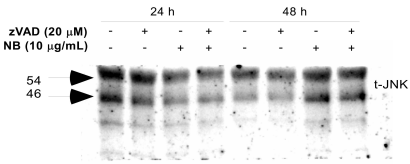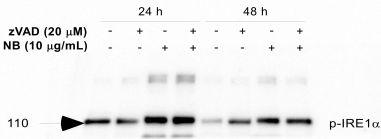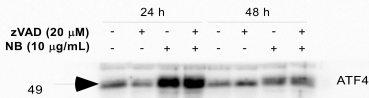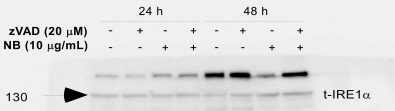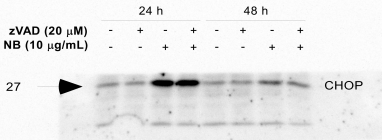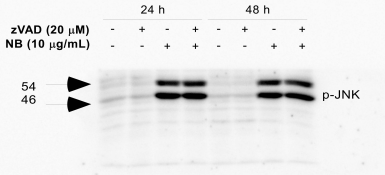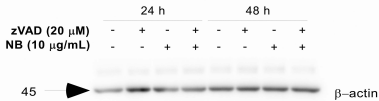

FIGURE 3 f

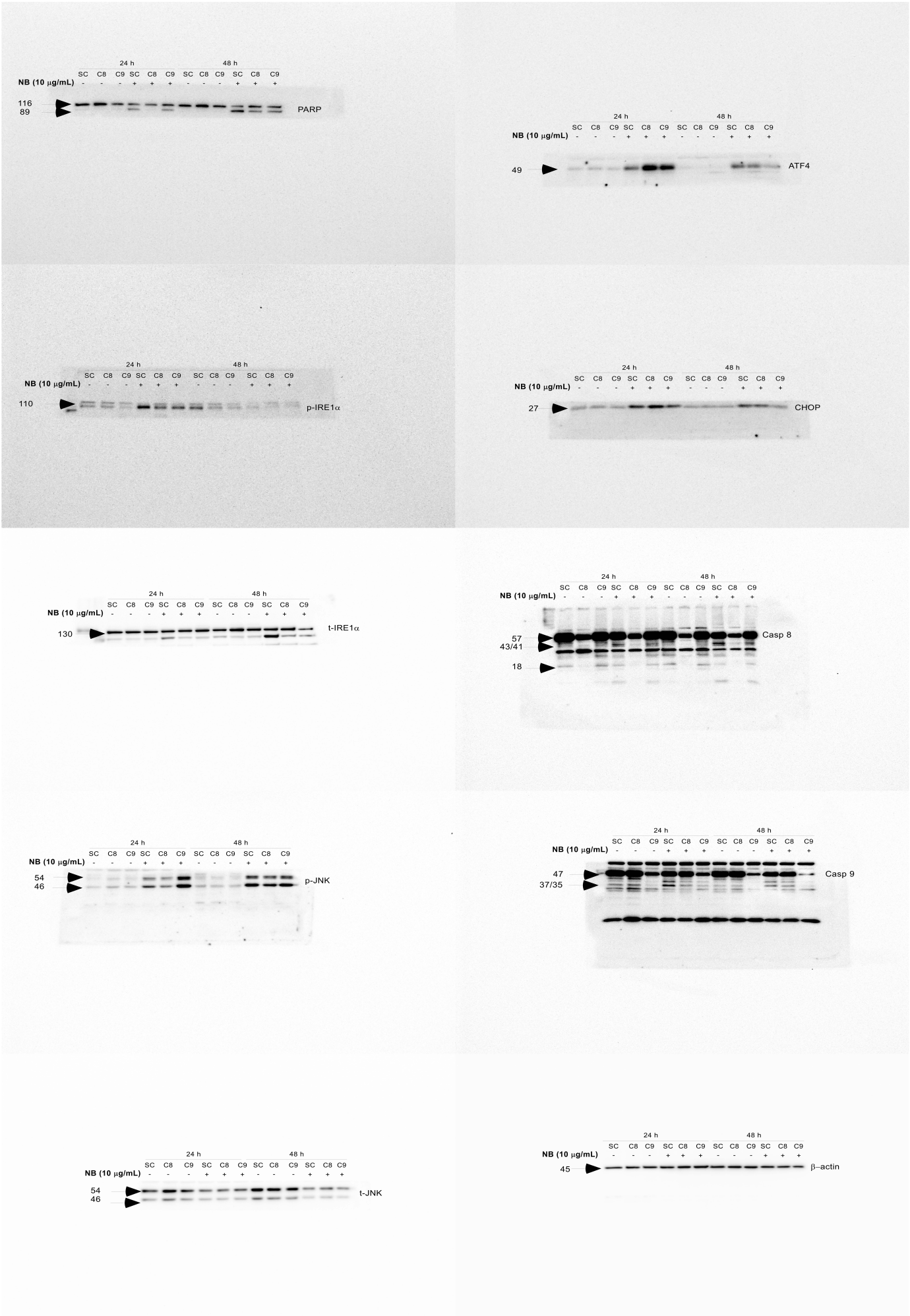

FIGURE 4 a

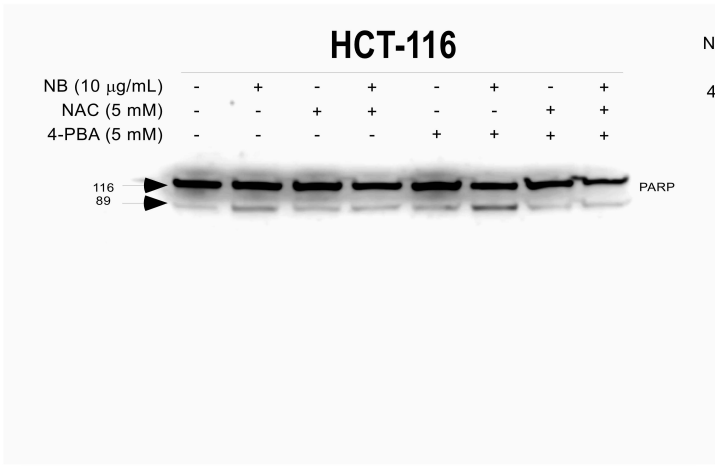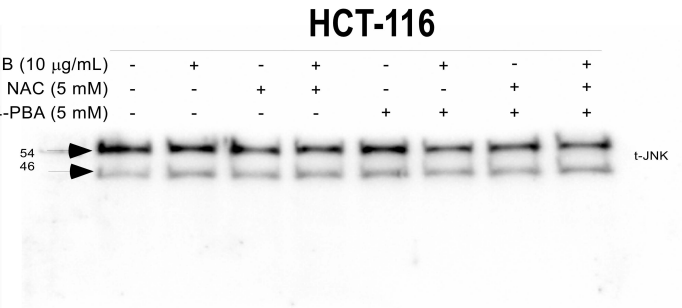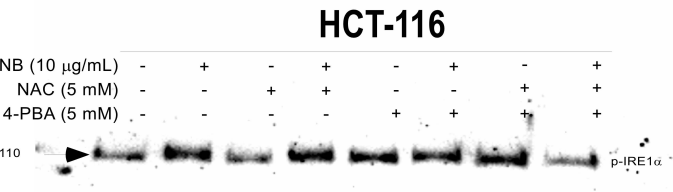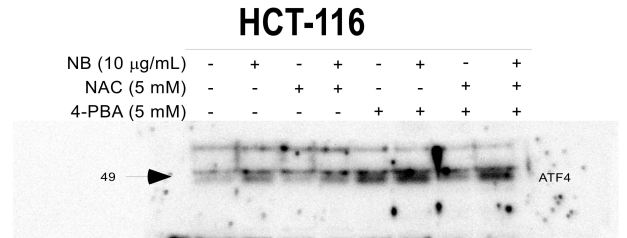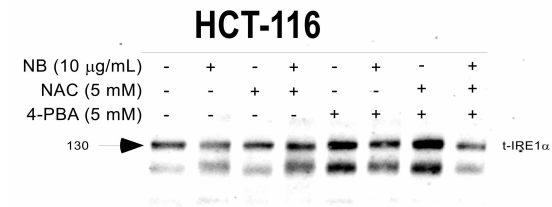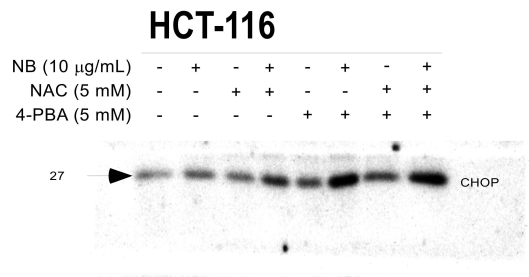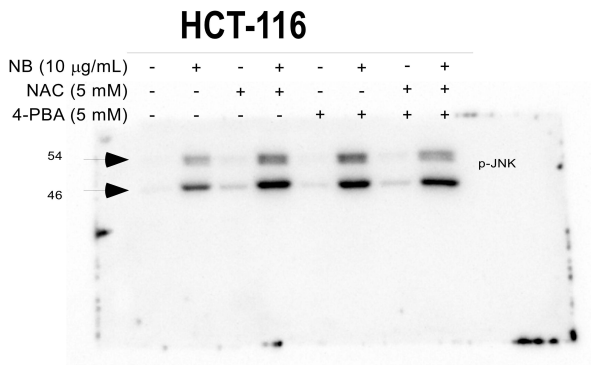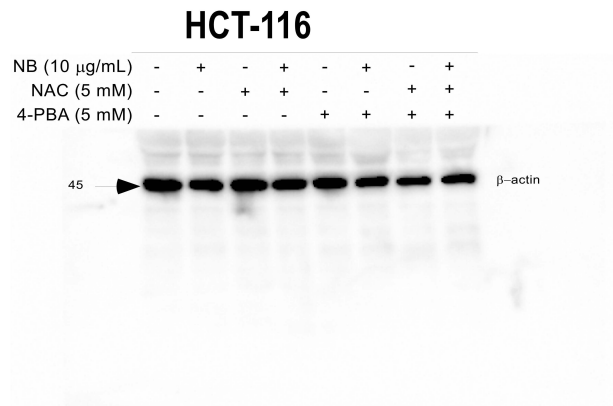

FIGURE 5 f

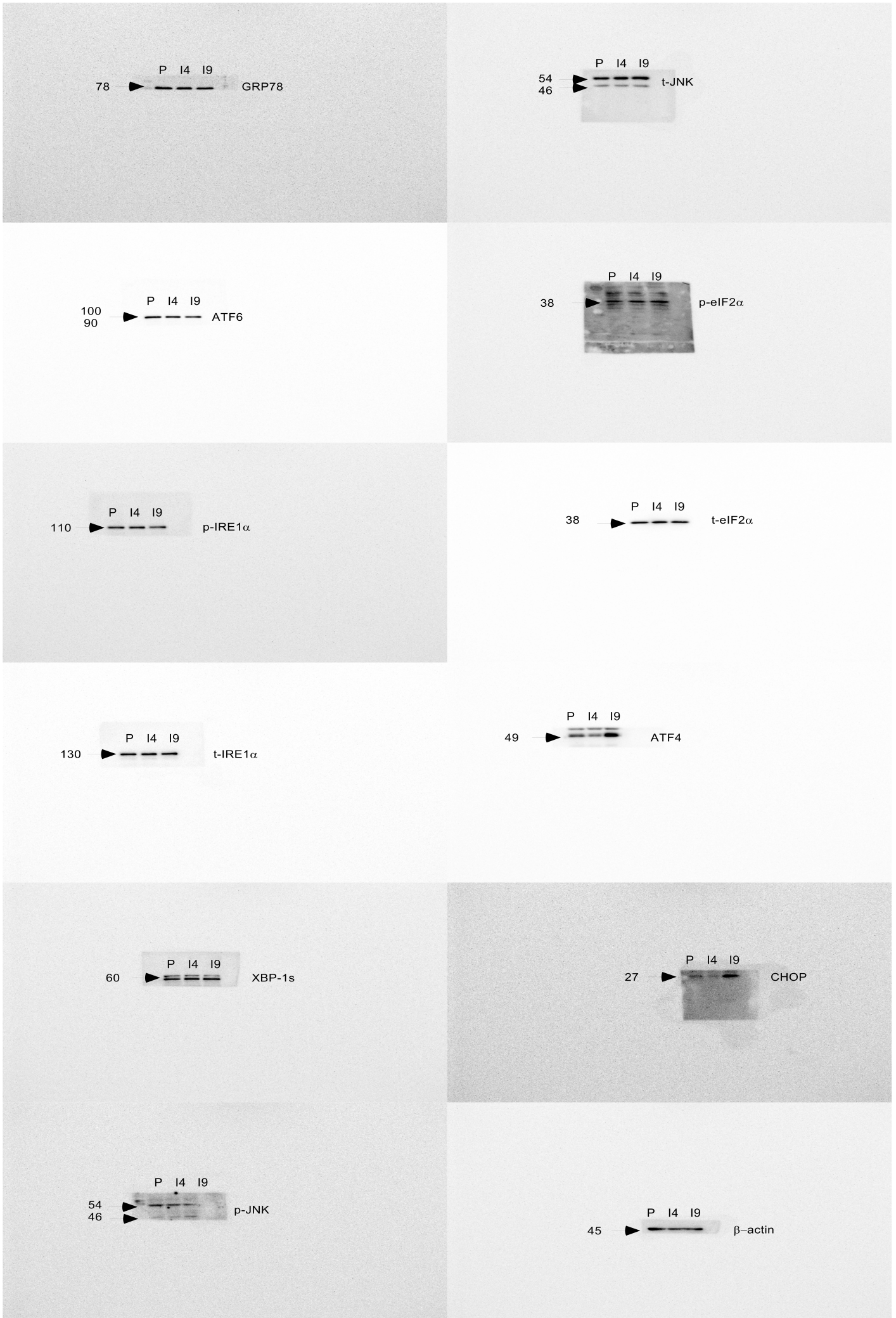

FIGURE 6 e

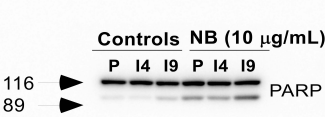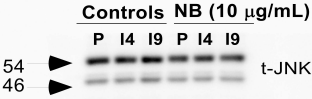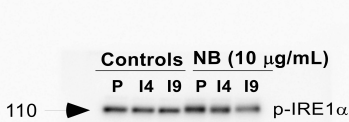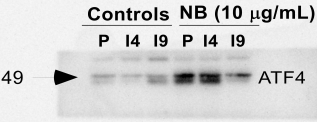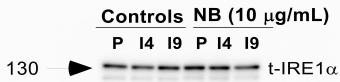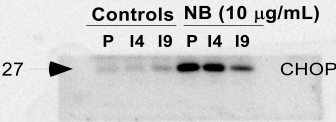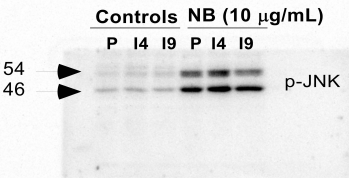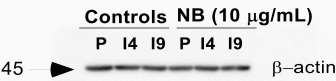

FIGURE 6 f

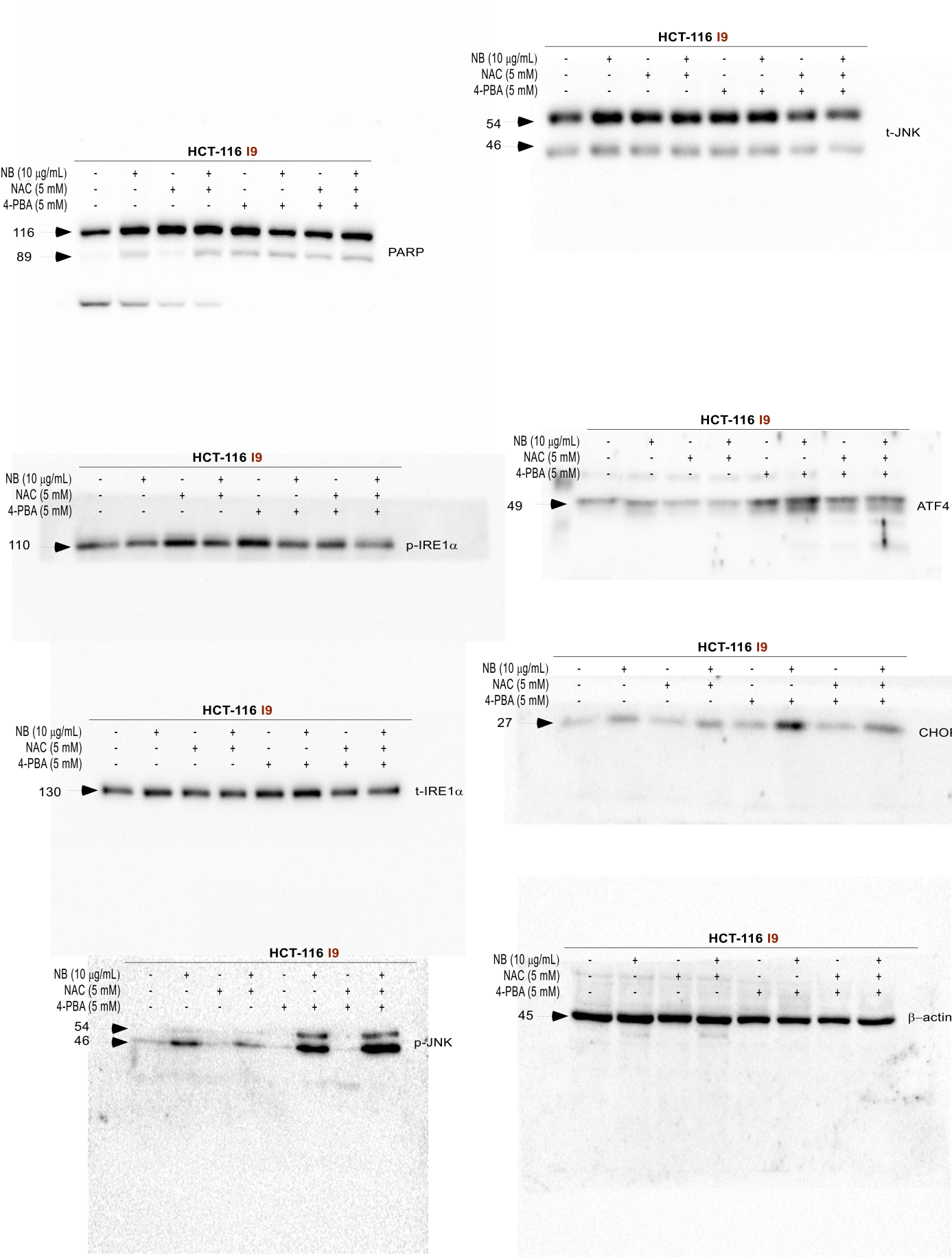

Supplementary FIGURE 7

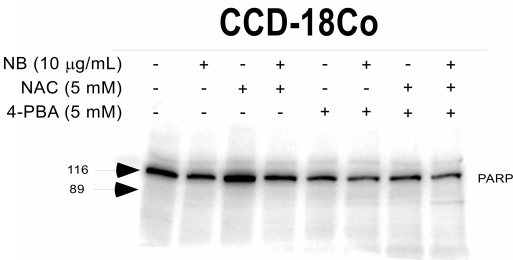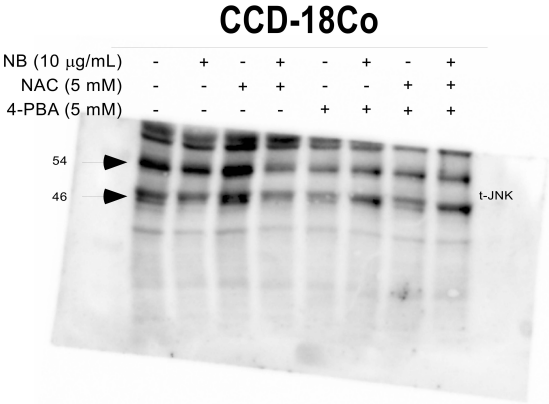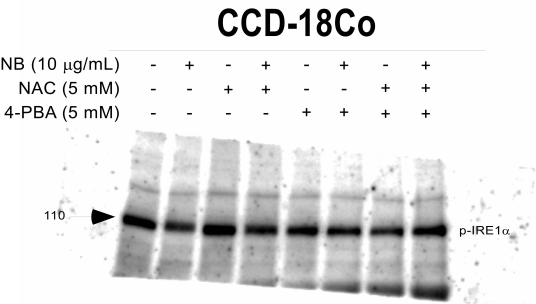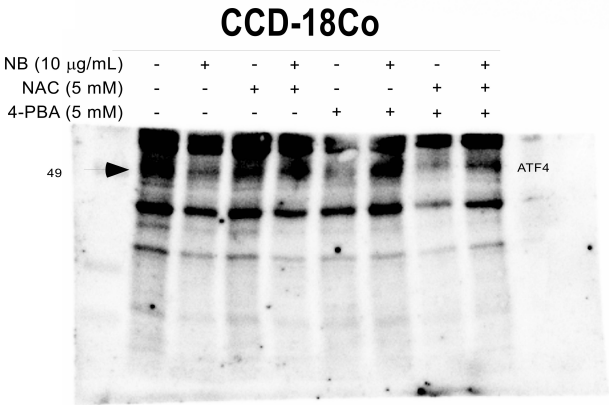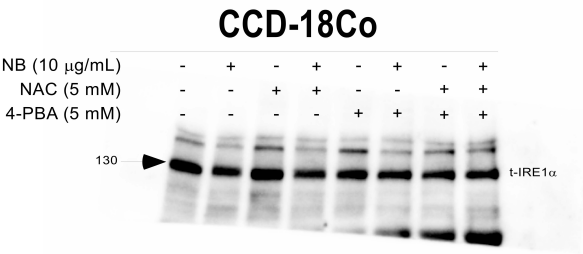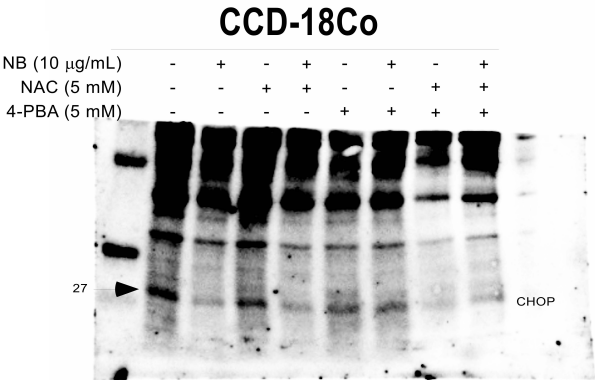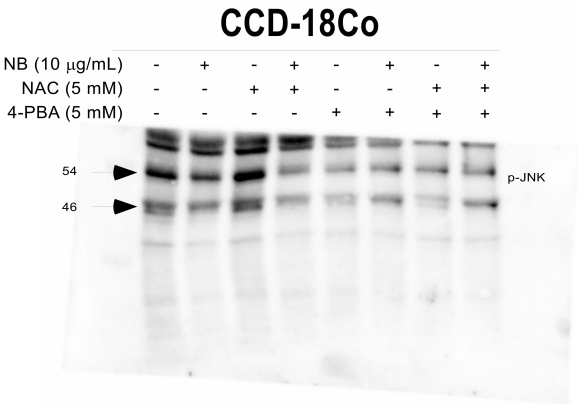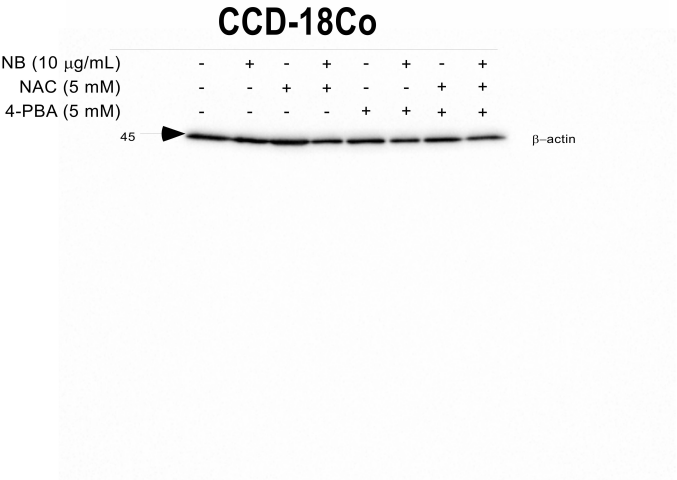

Supplement: Supplementary file 1 [file datasheet1.zip › Additional/IJMS_Blots.pdf]
